# Comparison with healthy controls: meta-analysis of changes in intestinal flora in Chinese patients with irritable bowel syndrome

**DOI:** 10.3389/fcimb.2025.1657501

**Published:** 2025-10-03

**Authors:** Hao Tu, Jingwen Xu, Liping Wei, Ziting Kong, Lun Cai

**Affiliations:** ^1^ Department of Neurology, The First Affiliated Hospital of Guangxi University of Chinese Medicine, Guangxi University of Chinese Medicine, Nanning, China; ^2^ Department of Rehabilitation, The First Affiliated Hospital of Guangxi University of Chinese Medicine, Guangxi University of Chinese Medicine, Nanning, China

**Keywords:** irritable bowel syndrome, intestinal flora, gut microbiota, Chinese, China, meta – analysis

## Abstract

**Background:**

The characteristics of the gut microbiota in Chinese patients with irritable bowel syndrome (IBS) and their association with the disease remain unclear. Although current studies have revealed some changes in the gut microbiota of IBS patients, these changes vary across different studies and no consistent conclusions have been reached. Additionally, geographical differences may lead to unique microbial characteristics in IBS patients, which could serve as potential diagnostic tools or therapeutic targets. We hypothesize that the gut microbiota of Chinese IBS patients exhibits unique compositional and functional characteristics that differ significantly from those of healthy individuals.

**Aim:**

To investigate differential characteristics of gut microbiota in Chinese patients with IBS compared to healthy controls (HC).

**Methods:**

We conducted a comprehensive search in PubMed, Web of Science, Embase, and China National Knowledge Infrastructure (CNKI) databases from their inception to May 2025. The search strategy was built around key concepts of IBS, gut microbiota, and China, combining subject headings (e.g., MeSH terms) and free-text keywords. The search was performed in line with the PRISMA guidelines. Studies were included if they were case-control studies comparing gut microbiota between IBS patients and all participants were Chinese, IBS diagnosis adhered to internationally recognized Rome criteria, HCs were free from gastrointestinal diseases, and sufficient data were provided for meta-analysis. Exclusion criteria encompassed non-case-control designs, studies involving non-Chinese populations or mixed control groups, unavailable or insufficient data, and studies of low methodological quality (NOS score < 5). Study selection was carried out by two researchers independently. Data were extracted and quality assessed using the Newcastle-Ottawa Scale (NOS). The quality assessment was independently conducted by two researchers, and any discrepancies were resolved with the assistance of a third researcher. Data were analyzed using RevMan 5.4 software, with standardized mean differences (SMD) and 95% confidence intervals (CIs) calculated. The I^2^ statistic was used to assess heterogeneity among studies. When I^2^ > 50%, indicating significant heterogeneity, the random effects model was used for calculations. Otherwise, the fixed effects model was applied.

**Results:**

This meta-analysis of 12 studies (766 IBS patients, 351 HC) revealed that Chinese IBS patients exhibited significantly higher abundance of *Enterobacter* (SMD = 1.55; 95% CI=[0.90,2.19]; *P* < 0.00001) and *Enterococcus* (SMD = 0.69; 95% CI=[0.25, 1.13]; *P* = 0.002), but lower *Lactobacillus* (SMD=-1.30; 95% CI=[-1.71, -0.89]; *P* < 0.00001) and *Bifidobacterium* (SMD=-1.56; 95% CI=[-2.04, -1.09]; *P* < 0.00001) versus HC, with no *Bacteroides* difference. In IBS-D Patients, *Enterobacter* (SMD = 1.47; 95% CI=[0.75, 2.19]; *P* < 0.0001) and *Enterococcus* (SMD = 0.53; 95% CI=[0.06, 1.01]; *P* = 0.03) remained elevated, while *Lactobacillus* (SMD=-1.39; 95% CI=[-1.76,-1.02];*P* < 0.00001), *Bifidobacterium* (SMD=-1.66; 95% CI=[-2.15,-1.17];*P* < 0.00001), and *Bacteroides* (SMD=-0.72; 95% CI=[-1.05, -0.39]; CI=[-1.05,-0.39];*P* < 0.0001) decreased significantly. IBS-C patients showed no significant differences in these genera.

**Conclusion:**

This meta-analysis reveals gut microbiota imbalances in Chinese IBS patients: significantly increased *Enterobacter* and *Enterococcus* with decreased *Lactobacillus* and *Bifidobacterium* overall, and reduced *Bacteroides* specifically in IBS-D.

## Introduction

Irritable Bowel Syndrome (IBS) is a functional gastrointestinal disorder characterized by recurrent abdominal pain, bloating, and altered bowel habits (Saha, 2014). It is classified into four subtypes: diarrhea-predominant (IBS-D), constipation-predominant (IBS-C), mixed (IBS-M), and unclassified (IBS-U) (Mearin et al., 2016). Epidemiological studies indicate that IBS-D accounts for approximately one-third of all IBS cases (Canavan et al., 2014). In China, the prevalence of IBS ranges from 1.4% to 11.5%, and this figure is increasing due to dietary and lifestyle changes (XL et al., 2025; Li and Li, 2015). This rising prevalence imposes a significant psychological and economic burden on patients (Di Rosa et al., 2023; Kosako et al., 2018). For instance, the annual direct economic cost of IBS treatment in China is approximately two billion US dollars (Zhang et al., 2016).

The Gut-Brain Axis (GBA), a complex bidirectional communication system between the gut and the brain, plays a crucial role in IBS’s pathophysiology (Sebastián Domingo, 2022). Factors like stress, psychological changes, intestinal flora imbalance, increased visceral sensitivity, and impaired intestinal mucosal barrier function can disrupt GBA function, inducing or exacerbating IBS symptoms (Yoshioka et al., 2022; Matsumoto et al., 2021). Stress, for instance, can disrupt the intestinal barrier’s integrity, triggering local immune responses and enhancing intestinal neuron excitability (Mayer et al., 2022; Jones et al., 2020). Animal experiments have confirmed that stress induces neuroendocrine abnormalities and mast cell hyperplasia in mice (Machorro-Rojas et al., 2019), which in turn affects intestinal motor function and visceral afferent nerve sensitivity (Hillestad et al., 2022). These pathological alterations induce heightened sensitivity of visceral afferent nerves, enteric nerves and enteric nervous system by transmitting to the nociceptors, which ultimately leads to intestinal dysfunction and the development of IBS symptoms (Mayer et al., 2014).

The Microbiota-Gut-Brain Axis theory posits that gut microbiota is essential for bidirectional communication between the enteric and central nervous systems. It modulates brain-gut signaling pathways and influences neurological outputs (H and L, 2023). Evidence indicates that gut microbiota critically regulates the hypothalamic-pituitary-adrenal (HPA) axis, whose normal development depends on beneficial bacterial colonization (Fan et al., 2022). In a mouse model of Tourette syndrome, fecal microbiota transplantation (FMT) intervention significantly increased serum 5-HT levels and promoted functional remodeling of the central nervous system (Li et al., 2022). Additionally, animal studies demonstrate that proximal colonic infusion of short-chain fatty acids (SCFAs) specifically activates (5-HT3R) receptors, enhancing colonic motility and triggering 5-HT release from enterochromaffin cells (Liu et al., 2021). These findings collectively suggest that gut microbiota rectifies neurofunctional abnormalities via central 5-HT metabolic network modulation (Kennedy et al., 2014). This multifactorial interplay elucidates how gut dysbiosis initiates IBS pathology, propagating systemic dysfunction through neuroendocrine-immune network dysregulation.

Geographic differences significantly affect the pathogenesis of IBS and the pattern of intestinal flora dysbiosis. Multiple factors such as environmental characteristics, dietary structure and lifestyle in different regions synergistically shape the characteristics of gut microbiota, which in turn modulates the epidemiological features and clinical phenotype of IBS. Studies have confirmed that diet contributes 57% to the shaping of gut microbiota structure, which is significantly higher than genetic factors (<12%). Regional dietary differences, such as high-sugar and high-fat Western dietary patterns, can induce dysbiosis and disrupt gastrointestinal metabolic and immune homeostasis (Sekirov et al., 2010). Additionally, environmental exposures (e.g., climate characteristics and soil microbial diversity) are positively correlated with gut microbiota diversity. Residents in areas with higher soil microbial abundance not only have more diverse gut microbiota but also have significantly lower IBS prevalence (Aggeletopoulou and Triantos, 2024). These geographic-specific factors drive microbial imbalance through multiple pathways, ultimately increasing the risk of IBS.

Although the association between gut microbiota alterations and irritable bowel syndrome (IBS) has been extensively documented globally, comprehensive and well-validated gut microbial biomarkers specific to the Chinese IBS population have not yet been fully established. Geographical variations may contribute to unique microbial signatures in Chinese patients, which could serve as potential biomarkers for IBS and its subtypes. Therefore, this study aims to systematically assess differences in gut microbiota between Chinese IBS patients and healthy controls, in order to elucidate the structure of the flora and the potential roles of specific taxa in the pathogenesis of IBS in China, thereby informing targeted therapeutic strategies.

## Materials and methods

### Protocol registration

The objectives and methodologies of this meta-analysis were predefined in a protocol registered with PROSPERO (Page et al., 2021). The registration was accepted on September 06, 2024, under the number CRD42024587766.

### Search methodology

We conducted a comprehensive search in PubMed, Web of Science, Embase, and China National Knowledge Infrastructure (CNKI) databases from their inception to May 2025. The search strategy was developed using a combination of controlled vocabulary terms (e.g., MeSH in PubMed) and keywords related to three core concepts: irritable bowel syndrome, gastrointestinal microbiota, and China. All retrieved records were imported into EndNote (version 20.5) for management. We included publications in both Chinese and English.

### Literature inclusion criteria

(1)Study design: Case-control studies comparing the gut microbiota between irritable bowel syndrome (IBS) patients and healthy controls (HCs). (2)Participants: Both IBS patients and healthy controls (HCs) must be Chinese. Patients in the case group must be diagnosed according to internationally recognized Rome criteria. HCs must be free from IBS and other gastrointestinal diseases. There were no restrictions on the gender or age of participants. (3) Data availability: Studies must provide sufficient data to calculate the standardized mean difference (SMD) and 95% confidence interval (CI) (e.g., means and standard deviations), or provide data that could be converted for such use. (4) Publication type: Full-text, original articles published in peer-reviewed journals in either English or Chinese.

### Exclusion criteria

(1)Ineligible study design: Non-case-control studies, including but not limited to: animal studies, reviews, meta-analyses, commentaries, case reports, conference abstracts, cross-sectional studies, cohort studies, and interventional studies (e.g., randomized controlled trials). (2)Ineligible population: Studies conducted in non-Chinese populations; or studies where not all participants were Chinese; or studies where the control group included patients with other gastrointestinal diseases (e.g., inflammatory bowel disease). (3)Data issues: Unretrievable full text, duplicate publications, or incomplete/unextractable data for meta-analysis. (4)Low quality: Studies judged to be of low methodological quality based on the Newcastle-Ottawa Scale (NOS) assessment (typically, a total score < 5).

### Literature screening and data extraction

Two researchers employed the same search strategy. Initially, the retrieved literature was imported into EndNote software for deduplication to remove duplicate articles. Subsequently, a preliminary screening was conducted by reviewing the titles and abstracts to exclude studies that were irrelevant to the research topic or did not meet the grouping criteria of this study. Finally, the full texts of the remaining articles were reviewed to select and include those that met the inclusion criteria. Any disagreements were resolved with the assistance of a third researcher. The primary content extracted from the studies included the first author’s name, publication year, diagnostic criteria, region, sample size, mean age, gender ratio, IBS subtype, names of the main gut microbiota studied, and the expression of gut microbiota abundance: including the mean, median, standard deviation, interquartile range, and *p*-values.

### Quality assessment

The quality of the included studies was assessed using the Newcastle-Ottawa Scale (NOS), a widely used tool for evaluating the quality of non-randomized studies (Stang, 2010). The NOS evaluates studies across three domains: selection of study groups, comparability of groups, and ascertainment of exposure or outcome. Each study can receive a maximum of 9 points, with higher scores indicating better quality; studies scoring 6 points or above were included in the data analysis. The quality assessment was independently conducted by two researchers, and any discrepancies were resolved with the assistance of a third researcher (**Table 1**).

**Table 1:** Methodological quality scores of the 12 included case-control studies according to the Newcastle-Ottawa Scale.

First author	Year	Selection	Comparability	Exposure	Total
Huang et al. (2023)	2023	4	2	2	8
Jiang et al. (2006)	2006	4	2	2	8
Si et al. (2004)	2004	4	2	2	8
Sun et al. (2021)	2021	3	2	2	7
Wu et al. (2019)	2019	4	2	3	9
Zhang et al. (2019)	2019	4	2	3	9
Zhao et al. (2021)	2021	4	2	2	8
Zhang et al. (2009)	2009	3	2	2	7
Zhuang et al. (2018)	2018	3	2	3	8
Zhuang et al. (2005)	2005	3	2	2	7
Ng et al. (2013)	2013	4	2	2	8
Su et al. (2018)	2018	4	2	3	9

### Data analysis

Review Manager (RevMan version 5.4) was used for data analysis. The abundance levels of gut microbiota in fecal and tissue samples from IBS patients were extracted. Mean differences (MDs) and 95% confidence intervals (CIs) were calculated as summary statistics for the abundance of gut microbiota obtained directly or indirectly from the included studies. The *I²* statistic was used to assess heterogeneity among studies. When *I²* > 50%, indicating significant heterogeneity, the random effects model was used for calculations. Otherwise, the fixed effects model was applied.

## Results

### Search results

**Figure 1:**
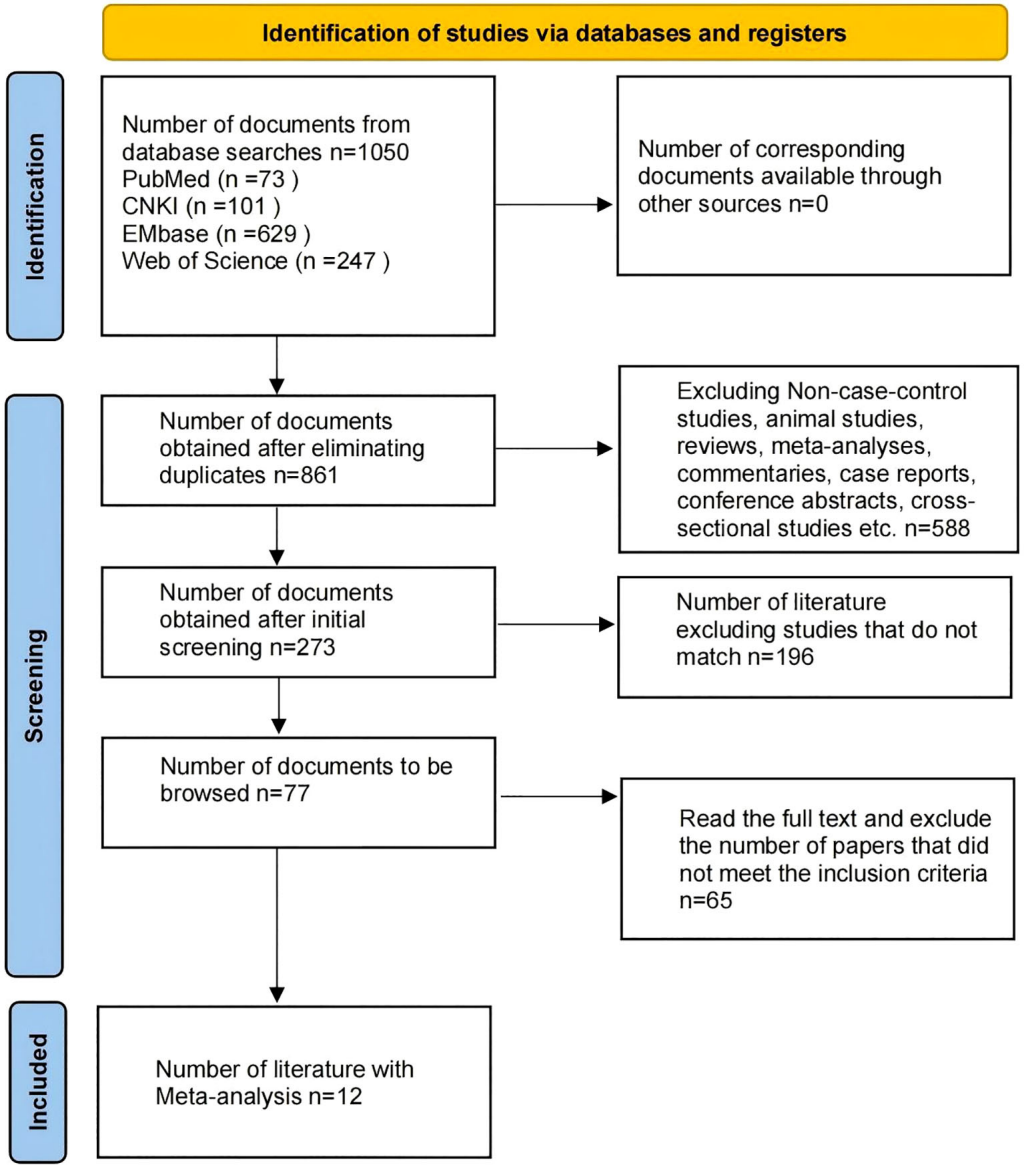
Flowchart of literature screening.

A total of 1,050 articles were identified through database searches, including 73 from PubMed, 101 from CNKI, 629 from Embase, and 247 from Web of Science. After removing duplicates using EndNote, 861 articles remained. The initial screening excluded reviews, meta-analyses, commentaries, letters, animal studies, and articles not published in Chinese or English, leaving 273 articles. After reviewing the titles and abstracts, 77 articles remained that met the inclusion criteria. Following full-text review, 65 articles were excluded for not meeting the study requirements, resulting in a final selection of 12 articles for the meta-analysis (**Figure 1**). All included studies were case-control studies, with a total sample size of 1,117, comprising 766 IBS patients and 351 HC.

### Basic characteristics of the included studies

A total of 12 studies were included, involving 766 IBS patients and 351 HC. These studies were published between 2004 and 2023, and all included patients had IBS (IBS-D, IBS-C), with diagnostic criteria ranging from Rome II to Rome IV. Ten studies obtained gut microbiota data from fecal samples, while two studies used tissue samples. Seven studies utilized 16S rRNA sequencing to assess microbial abundance. Additionally, five studies evaluated microbial abundance using culture methods. For detailed information (**Table 2**).

**Table 2:** Characteristics and methods of the 12 studies.

First author	Year	Location	IBS Diagnosis	n IBS/HC	Age,IBS (range ,x ± s)	Ratio of men to women	Experimental Methods	Sample	Cited material
Huang et al.	2023	Haian	RomeIII	93/30	31.59 ± 4.15 32.61 ± 5.31/ 31.87 ± 5.15	57/66	Culture	Tissue	(Huang and Ding, 2023)
Jiang et al.	2006	Guangzhou	Rome II	43/25	33.62 ± 8.29 32.27 ± 8.63/ 29.52 ± 7.83	35/33	Culture	Stools	(Jiang et al., 2006)
Si et al.	2004	Hangzhou	RomeII	25/25	45.40 ± 10.56/ 45.40 ± 10.56	16/34	Culture	Stools	(Si et al., 2004)
Sun et al.	2021	Beijing	Rome III	162/66	35.53 ± 11.72/ 36.67± 11.83	147/81	16S rRNA sequencing	Stools	(Sun et al., 2021)
Wu et al.	2019	Chongqing	Rome IV	60/60	/	77/43	16S rRNA sequencing	Stools	(Wu et al., 2019)
Zhang et al.	2019	Nanjing	Rome III	70/30	37.17 ± 14.37 36.74 ± 15.11/ 39.10 ± 12.09	51/49	16S rRNA sequencing	Stools	(Zhang et al., 2019)
Zhao et al.	2021	Shanghai	Rome III	152/30	56.38 ± 7.13 57.02 ± 8.06 56.07 ± 7.58/ 57.11 ± 8.06	121/61	Culture	Stools	(Zhao and Wang, 2021)
Zhang et al.	2009	Beijing	Rome III	50/25	/	/	16S rRNA sequencing	Stools	(Zhang et al., 2009)
Zhuang et al.	2018	Guangzhou	Rome III	27/13	32.1 ± 8.11/ 30.54 ± 6.75	/	16S rRNA sequencing	Stools	(Zhuang et al., 2018)
Zhuang et al.	2005	Mudanjiang	Rome II	34/17	43.27 ± 13.73 42.81 ± 16.61 41.83 ± 11.75/ 43.24 ± 9.50	21/30	Culture	Stools	(Zhuang et al., 2005)
Ng et al.	2013	Hong Kong	Rome III	10/10	45.5 ± 18.52/ 46 ± 18.52	4/16	16S rRNA sequencing	Tissue	(Ng et al., 2013)
Su et al.	2018	Hangzhou	Rome III	40/20	/	/	16S rRNA sequencing	Stools	(Su et al., 2018)

### Quality assessment of included studies

The methodological quality of the 12 included case-control studies was evaluated using the Newcastle-Ottawa Scale (NOS). NOS scores ranged from 7 to 9 (maximum 9) with a mean of 8.0 ± 0.7 SD, indicating high overall quality. All studies employed strict diagnostic criteria for IBS cases and defined healthy controls, with comparability adjustments for key confounders including age and sex. Point deductions primarily occurred in two domains: Selection: (Sun et al., 2021) and (Zhang et al., 2009) lost points due to non-concurrent control sourcing (community/WeChat-recruited controls vs. hospital-sourced IBS cases), while (Zhuang et al., 2005) failed to explicitly exclude IBS in controls. Exposure: All studies scored full points for standardized microbiome protocols (Item F). Crucially, Item G (blinding) was uniformly awarded 1 star as group awareness is methodologically necessary for differential microbiome analysis in case-control comparisons - enforced blinding would introduce analytical bias during bioinformatic processing. However (Sun et al., 2021; Zhang et al., 2009; Zhuang et al., 2005) lost points for unreported sample exclusion rates/data completeness (Item H).

### Meta-analysis results

This study conducted a statistical analysis of gut microbiota. In the included studies, there were significant differences in gut microbiology between individuals with IBS and HC (*P* < 0.05), with a significant difference between IBS-D and HC (*P* < 0.05), while there was no significant difference in IBS-C compared to HC (*P* > 0.05). The analysis focused primarily on the genus level. To reduce bias, the relative abundance of gut microorganisms measured in at least 12 studies was calculated. The relative abundance, mean and standard deviation of microorganisms in the genera *Enterobacter*, *Enterococcus*, *Bacteroides*, *Lactobacillus*, and *Bifidobacterium* were determined. These microorganisms were discussed in depth in the included studies.

**Figure 2:**
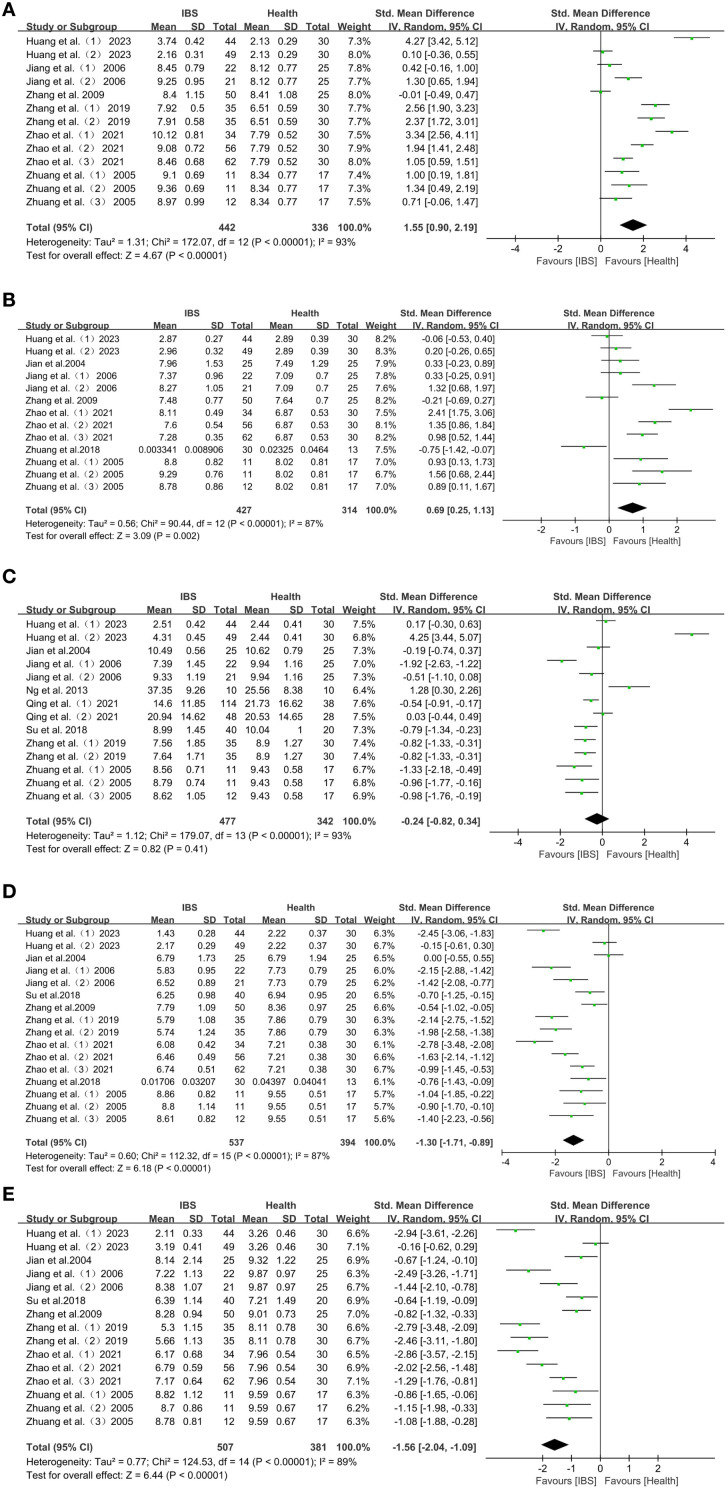
**(A)** Forest plot of changes in *Enterobacter* in IBS patients with HC. **(B)** Forest plot of changes in *Enterococcus* in IBS patients with HC. **(C)** Forest plot of changes in *Bacteroides* in IBS patients with HC. **(D)** Forest plot of changes in *Lactobacillus* in IBS patients with HC. **(E)** Forest plot of changes in *Bifidobacterium* in IBS patients with HC.

Overall, the forest plot of this study showed that in Chinese IBS patients, compared with HC individuals, the abundance of *Enterobacter* (SMD = 1.55; 95% CI = [0.90, 2.19]; *P* < 0.00001) (**Figure 2A**), as well as *Enterococcus* (SMD = 0.69; 95% CI = [0.25, 1.13]; *P* = 0.002) (**Figure 2B**), were elevated in abundance and significantly reduced in *Lactobacillus* (SMD = -1.30; 95% CI = [-1.71, -0.89]; *P* < 0.00001) (**Figure 2D**), and *Bifidobacterium* (SMD = -1.56; 95% CI = [-2.04, -1.09]; *P* < 0.00001) (**Figure 2E**). In contrast, the abundance of *Bacteroides* (SMD = -0.24; 95% CI = [-0.82, 0.34]; *P* = 0.41), was not significantly different from that of HC individuals (**Figure 2C**).

**Figure 3:**
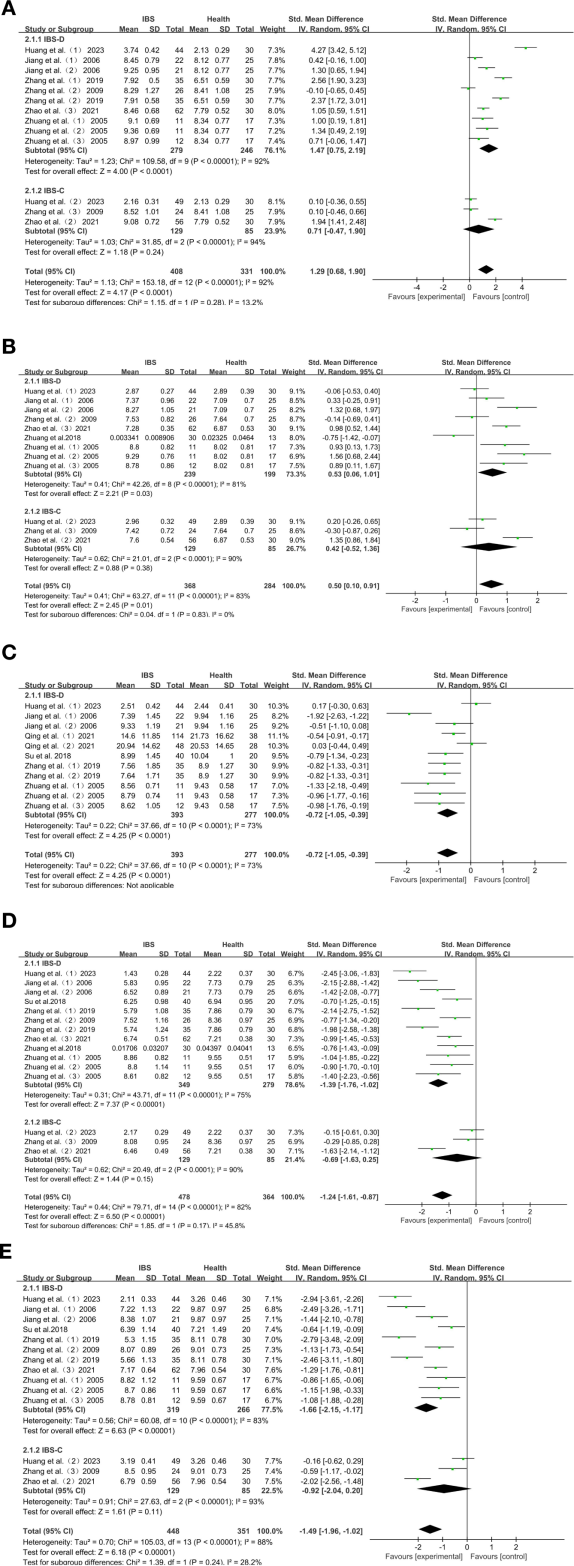
**(A)** Forest plot of IBS-D, IBS-C and HC intestinal flora change subgroup analyses: *Enterobacter.* **(B)** Forest plot of IBS-D, IBS-C and HC intestinal flora change subgroup analyses: *Enterococcus.* **(C)** Forest plot of IBS-D, IBS-C and HC intestinal flora change subgroup analyses: *Bacteroides.* **(D)** Forest plot of IBS-D, IBS-C and HC intestinal flora change subgroup analyses: *Lactobacillus.* **(E)** Forest plot of IBS-D, IBS-C and HC intestinal flora change subgroup analyses: *Bifidobacterium.* Egger’s test: *P* = 0.024.

There were significant differences in the characteristics of intestinal flora changes in Chinese patients with IBS-D. The characteristics of intestinal flora changes in patients with IBS-D were: increased abundance of *Enterobacter* and *Enterococcus*: *Enterobacter* (SMD = 1.47; 95% CI = [0.75,2.19]; *P* < 0.0001) (**Figure 3A**), and *Enterococcus* (SMD = 0.53; 95% CI = [0.06, 1.01]; *P* = 0.03) (**Figure 3B**). *Lactobacillus*, *Bifidobacterium* and *Bacteroides* were reduced in abundance: *Lactobacillus* (SMD = -1.39; 95% CI = [-1.76, -1.02]; *P* < 0.00001) (**Figure 3D**), *Bifidobacterium* (SMD = -1.66; 95% CI = [-2.15, -1.17]; *P* < 0.00001) (**Figure 3E**), *Bacteroides* (SMD=-0.72; 95% CI = [-1.05, -0.39]; *P* < 0.0001) (**Figure 3C**).

Among Chinese IBS-C patients, *Enterobacter* (SMD = 0.71; 95% CI = [-0.47,1.90]; *P* = 0.24) (**Figure 3A**), *Enterococcus* (SMD = 0.42; 95% CI = [-0.52,1.36]; *P* = 0.38) (**Figure 3B**), *Lactobacillus* (SMD = -0.69; 95% CI = [-1.63,0.25]; *P* = 0.15) (**Figure 3D**), *Bifidobacterium* (*SMD* = -0.92; 95% CI = [-2.04, 0.20]; *P* = 0.11) (**Figure 3E**), were not significantly different compared to HC.

### Subgroup analysis

In order to investigate the microbial variation in the different subtypes of IBS, we analyzed *Enterobacter*, *Enterococcus*, *Bacteroides*, *Lactobacillus* and *Bifidobacterium*, in the different subtypes of IBS. Other bacteria were not included because they were analyzed as subtypes in less than three studies. The expression of each subtype was compared with HC. Subgroup analyses showed that *Enterobacter* (SMD = 1.47; *P* < 0.0001), and *Enterococcus* (SMD = 0.53; *P* = 0.03), were more abundant than in HC in Chinese patients with IBS-D, while *Lactobacillus* (SMD=-1.39; *P* < 0.00001), *Bifidobacterium* (SMD=-1.66; *P* < 0.00001), and *Bacteroides* (SMD=-0.72; *P* < 0.0001), were less abundant than in HC. The expression of *Enterobacter*, *Enterococcus*, *Lactobacillus*, and *Bifidobacterium* on the other hand, did not differ significantly in Chinese IBS-C patients.

### Publication bias assessment

**Figure 4:**
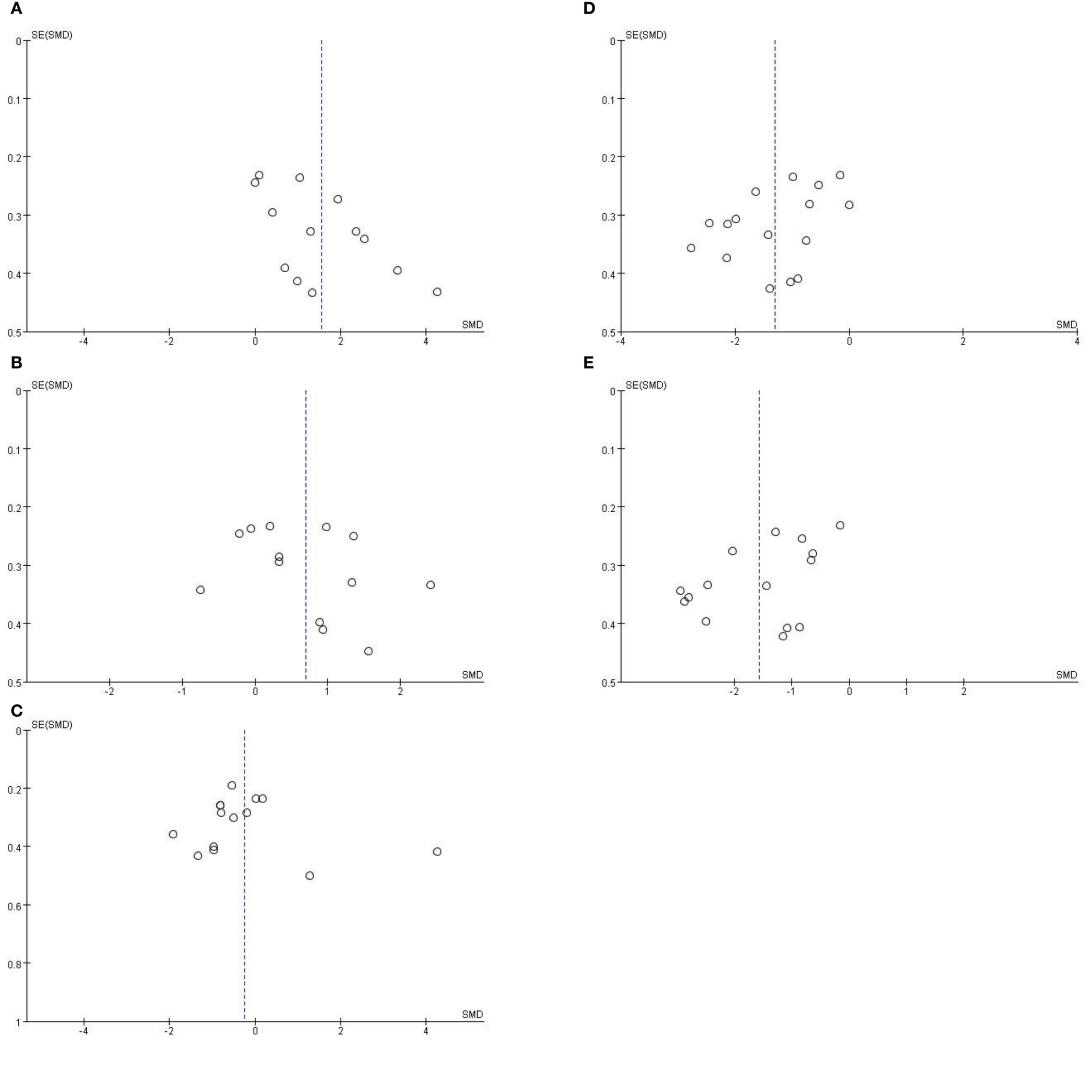
**(A)** Funnel plot assessing publication bias for *Enterobacter* abundance in IBS patients with HC. Egger’s test: *P* = 0.265. **(B)** Funnel plot assessing publication bias for *Enterococcus* abundance in IBS patients with HC. Egger’s test: *P* = 0.543. **(C)** Funnel plot assessing publication bias for *Bacteroides* abundance in IBS patients with HC. Egger’s test: *P* = 0.081. **(D)** Funnel plot assessing publication bias for *Lactobacillus* abundance in IBS patients with HC. Egger’s test: *P* = 0.048. **(E)** Funnel plot assessing publication bias for *Bifidobacterium* abundance in IBS patients with HC.

To evaluate potential publication bias, we conducted Egger’s regression tests alongside funnel plot analysis, accounting for the high heterogeneity observed across bacterial taxa (*I*² = 73–93%); the results revealed significant funnel plot asymmetry for *Enterobacter* (Egger’s test, *P* = 0.024; **Figure 4A**) and *Bifidobacterium* (Egger’s test, *P* = 0.048; **Figure 4E**), while no significant asymmetry was detected for *Enterococcus* (*P* = 0.265; **Figure 4B**), *Bacteroides* (*P* = 0.543; **Figure 4C**), or *Lactobacillus* (*P* = 0.081; **Figure 4D**). Crucially, this asymmetry is attributed to inherent clinical and methodological heterogeneity rather than selective publication bias: for *Enterobacter*, the directional bias aligns with its biologically meaningful enrichment in IBS-D patients (SMD = 1.47, *P* < 0.0001), confirming disease-subtype specificity and validating the robustness of this finding; for *Bifidobacterium*, the asymmetry coincided with extreme heterogeneity (*I*² > 85%) and stemmed from predefined sources of variation, including evolving diagnostic criteria (from Manning to Rome I–IV standards), methodological differences (16S rRNA sequencing vs. culture-based assays), sample-type discrepancies (fecal vs. mucosal specimens), unadjusted confounding factors (e.g., unassessed psychological status), and geographic variability across study populations. These multidimensional sources of heterogeneity explain the observed funnel plot asymmetry, indicating that publication bias did not materially affect the core conclusions of this meta-analysis.

## Discussion

### Study findings

This meta-analysis of 12 studies involving 766 IBS patients confirmed the significant association between intestinal dysbiosis and IBS. Specifically, Chinese IBS patients showed a significant decrease in beneficial flora such as *Lactobacillus* and *Bifidobacterium* in the intestine, while potentially pathogenic flora such as *Enterobacter* and *Enterococcus* were amplified, both (*P* < 0.05); notably, *Bacteroides* abundance did not show a statistically significant difference between groups (*P*> 0.05). Subgroup analyses showed that Chinese IBS-D patients were characterized by imbalanced flora: increased abundance of *Enterobacter* and *Enterococcus* and decreased abundance of *Lactobacillus*, *Bifidobacterium* and *Bacteroides* while the above flora in Chinese IBS-C patients did not show any significant difference from HC (*P*> 0.05).

### Comparison with previous studies

In this study, we conducted a geographically specific meta-analysis to delineate gut microbiota profiles in Chinese IBS patients versus healthy controls. Building upon previous research, our analysis incorporated a diverse range of microbial taxa and specifically examined five key bacterial genera, most of which demonstrated statistically significant alterations. Furthermore, we performed comprehensive subtype stratifications (IBS-D and IBS-C) to reveal subtype-specific microbial patterns. This approach provides a refined perspective on gut microbiota dysbiosis in Chinese IBS populations, supporting future development of targeted microbiota-based interventions.

### Altered intestinal flora in overall IBS patients

In comparison with previous meta-analyses by Wang et al. (Wang et al., 2020), which included 23 studies, and Zhuang et al. (Zhuang et al., 2017), which included 17 studies, each covering multi-geographic populations, the present study, along with these earlier works, confirms the existence of a core flora imbalance characterized by the proliferation of conditionally pathogenic bacteria (e.g., *Enterobacter*) and depletion of probiotic bacteria (e.g., *Lactobacillus* and *Bifidobacterium*) in IBS patients. Additionally, the present study, in agreement with the findings of Zhuang et al. (Zhuang et al., 2017), found no significant difference in the abundance of *Bacteroides* between IBS patients and healthy controls (*P* > 0.05). The points of difference were as follows: (1) Divergent findings regarding *Bacteroides* abundance: Wang et al. (Wang et al., 2020) reported significantly elevated *Bacteroides* abundance in Chinese IBS patients (*P* < 0.01); Zhuang et al. (Zhuang et al., 2017) found no statistically significant difference in its abundance (*P* > 0.05). Whereas the present study demonstrated marked reduction specifically in IBS-D subgroups (*P* < 0.0001). This discrepancy suggests that distributional differences of IBS subtypes could be a key factor modulating gut microbiota profiles. (2) Controversy over the IBS-C bacterial flora: Wang et al. (Wang et al., 2020) reported that subgroup analyses showed results consistent with those of untyped IBS analyses, whereas the intestinal flora of IBS-C patients in the present study was not significantly different from that of HC, a phenomenon that may be related to the insufficient data on the intestinal flora of the IBS-C patients included in the present study. (3) Differences in *Enterococcus* abundance: Wang et al. (Wang et al., 2020) and Zhuang et al. (Zhuang et al., 2017) both reported no statistically significant differences in *Enterococcus* abundance between IBS patients and healthy controls, whereas the present study identified a significantly higher abundance. While the former studies compared gut microbiota between Chinese and other geographic populations, neither specifically focused on alterations unique to Chinese IBS patients. In contrast, the current analysis exclusively targeted this subgroup. The resulting discrepancies may stem from variations in dietary habits, cultural practices, or geographic factors influencing microbiota composition. Together, the findings indicate that although gut dysbiosis in IBS exhibits certain consistent patterns across populations, subtype variations and geographic specificity warrant further investigation through standardized, multicenter studies.

### Altered intestinal flora in patients with IBS-D

In comparison with the meta-analysis by Liu et al. (Liu et al., 2017) the present study yielded both convergent and divergent findings. Consistent with their results, we confirmed a significant reduction in the abundance of *Lactobacillus* and *Bifidobacterium* in IBS-D patients (*P* < 0.05), reinforcing the notion that depletion of these probiotic genera represents a core feature of IBS-D–associated dysbiosis. However, several differences were also observed. Specifically, our analysis detected a significantly higher abundance of *Enterobacter* and *Enterococcus*, along with a marked reduction in *Bacteroides* in IBS-D patients—changes not reported in the earlier meta-analysis. These discrepancies may be attributable to variations in sample sources (e.g., geographic and dietary background), study inclusion criteria (e.g., symptom severity thresholds), or methodological approaches (e.g., 16S rRNA sequencing). Although both studies contribute evidence supporting microbiota dysbiosis in IBS, our study offers a more refined perspective through detailed subtype-focused analysis and an expanded range of microbial genera, thereby providing better-targeted microbial indicators for the development of future intervention strategies.

The growth of *Bifidobacterium* is closely linked to dietary fiber intake, and numerous plant polysaccharides have been shown to promote the metabolism and proliferation of *Bifidobacterium* (Kelly et al., 2021). According to the Report on Nutrition and Chronic Disease Status of Chinese Residents (2020), the modern dietary structure in China is generally characterized by high fat, high sugar, and low fiber, leading to inadequate dietary fiber intake, which further inhibits the growth of *Bifidobacterium.* In addition, the reduced intake of probiotic-rich fermented foods has also led to insufficient supplementation of *lactobacillus*. The use of antibiotics further exacerbates the imbalance of gut flora. For example, a study of the inhibitory effect of amoxicillin on *Bifidobacterium adolescentis* showed a significant decrease in the number of viable bacteria with increasing concentrations of amoxicillin, accompanied by disturbances in pathways such as arginine and proline metabolism, and glutathione metabolism (Wang et al., 2023). In addition, it was found that five strains of probiotics (including two strains of *Lactobacillus acidophilus* and three strains of *Bifidobacterium animalis*) exhibited varying susceptibility to acetylspiramycin, streptomycin, and penicillin (Fen et al., 2012), suggesting that antibiotic treatment may have a wide range of impacts on the intestinal probiotic flora. In summary, the combined effects of modern dietary patterns, antibiotic overuse, and inadequate probiotic consumption contribute to the reduction of *Bifidobacterium* and *Lactobacillus* in the intestinal flora of IBS-D patients.

*Bifidobacterium* helps resist colonization and invasion by enteric pathogens, enhances the intestinal epithelial barrier, and produces metabolites that strengthen gut defenses. This suggests that gut microbiota imbalance may trigger IBS (Binbin et al., 2016). *Lactobacillus* is crucial in intestinal immunomodulation and maintains healthy intestinal homeostasis through multiple mechanisms. On the one hand, it promotes secretory immunoglobulin A (SIgA) secretion and prevents pathogen adhesion and invasion, which is the key to intestinal mucosal immune defence. On the other hand, *Lactobacillus* plays a key role in intestinal immunomodulation and maintains homeostasis through multiple mechanisms. It promotes secretory immunoglobulin A (SIgA) secretion, preventing pathogen adhesion and invasion—a crucial aspect of mucosal immunity. Additionally, *Lactobacillus* regulates cytokines such as interleukin-2 (IL-2) and interferon-γ (IFN-γ). IL-2 activates T-lymphocytes and enhances cellular immunity, while IFN-γ exhibits antiviral, antibacterial, and antitumor properties, modulates immune cell activity, and helps maintain immune balance. Moreover, *Lactobacillus* is tolerant to acid and bile salts, metabolizes prebiotics to produce short-chain fatty acids, and supplies energy for beneficial gut bacteria, thereby supporting microbial structure and function. Thus, a decrease in *Lactobacillus* may disrupt flora balance, promote harmful bacteria, trigger intestinal inflammation and abnormal immune responses, and increase the risk of IBS (Runguo et al., 2017).

The present study clearly demonstrates a significant increase in the abundance of *Enterobacter* and *Enterococcus* in IBS patients. The widespread long-term use of antibiotics in healthcare, agriculture, and animal husbandry in China has significantly increased selective pressure on bacterial populations. Bacteria carrying drug resistance genes are more likely to survive and thrive, given their genetic advantages. Mechanistically, broad-spectrum antibiotics wipe out large numbers of drug-sensitive bacteria, while *Enterobacter* and *Enterococcus* face less inhibition due to intrinsic resistance mechanisms (W and L, 2008; Yan et al., 2024). For example, vancomycin-resistant enterococci (VRE) have emerged as a serious clinical challenge. VRE are resistant to many common antibiotics, limiting treatment options and complicating clinical management (Ahmed et al., 2023). Similarly, Enterobacter species can acquire resistance genes—such as plasmid-mediated quinolone resistance (PMQR) genes—via mobile genetic elements like plasmids and transposons (Cao et al., 2017). Through this process, resistant *Enterobacter* and *Enterococcus* gain a competitive advantage, enabling them to dominate the microbial community.

As a conditional pathogen, *Enterobacter* contributes to IBS pathogenesis through intestinal infection and flora imbalance. The colonization of pathogenic subtypes such as enterotoxigenic *Escherichia coli* (ETEC) can cause acute diarrhea, and recurrent infections may increase IBS risk by disrupting intestinal homeostasis (Fleckenstein and Kuhlmann, 2019). *Enterococcus*, on the other hand, may increase intestinal permeability by compromising mucosal integrity. This can lead to gastrointestinal dysmotility, visceral hypersensitivity, immune activation, and low-grade inflammation—all of which are closely associated with the development of IBS (L and T, 2018).

In this study, we synthesized data from multiple studies to identify potential factors contributing to the decreased abundance of *Bacteroides* in patients with IBS-D. (1) Irrational use of antibiotics: Cao Jingui et al. (Cao et al., 1995) and others have demonstrated that antibiotic misuse can significantly reduce the abundance of core commensal bacteria, such as *Bifidobacterium* and *Bacteroides*, while promoting the proliferation of conditional pathogens like *Enterobacter*. This disruption in microbial homeostasis can lead to pathological changes in the host. (2) Dietary abnormalities: High sugar intake: Studies indicate that a high-sugar diet can markedly increase the relative abundance of *Proteobacteria* while inhibiting the growth of *Bacteroides* (Satokari, 2020). High-fat diet: Duan Haoliang et al. (Duan Haoliang, 2023) showed through dietary intervention experiments that the abundance of *Bacteroides* was significantly lower in a high-fat diet group compared to a high-fiber diet group, suggesting that abnormal lipid metabolism suppresses *Bacteroides*. Collectively, this evidence implies that antibiotic abuse and high-sugar/high-fat diets may serve as exogenous factors reducing *Bacteroides* abundance. These factors likely contribute to intestinal disorders by disrupting flora structure and interfering with metabolic homeostasis.

In patients with IBS-D, *Bacteroides* aids the host in catabolizing polysaccharides to enhance nutrient utilization. It also promotes intestinal mucosal vascularization and supports immune system development, thereby improving host immunity and maintaining intestinal microecological balance. A decrease in *Bacteroides* abundance may lead to reduced nutrient absorption efficiency, impaired intestinal immune function, and disruption of microbial equilibrium, ultimately contributing to the pathogenesis of IBS-D (Zhu et al., 2021; Chuan et al., 2019).

### Altered gut flora in patients with IBS-C

The present study demonstrated that in patients with the constipation-predominant IBS-C subtype, the abundances of *Enterobacter*, *Enterococcus*, *Lactobacillus*, and *Bifidobacterium* did not differ significantly from those in HC. Current research on IBS-C remains limited, primarily due to small sample sizes and relatively narrow patient populations. It is recommended that future multi-center, large-sample studies be conducted to explore the pathological mechanisms of IBS-C in the Chinese population, thereby enhancing the evidence base for this subtype.

### Implications for clinical practice

This meta-analysis systematically revealed specific alterations in the gut microbiota of Chinese patients with IBS. The clinical relevance of these findings lies in the following two aspects: (1) Diagnostic value of microbial biomarkers: Research shows that the abundance of *Enterobacter* and *Enterococcus* in the gut of Chinese IBS patients is significantly higher than in healthy controls, while the abundance of *Lactobacillus* and *Bifidobacterium* is significantly reduced, and this study further identified a decreased abundance of *Bacteroides* specifically in IBS-D patients. This characteristic difference suggests that quantitative detection of the above bacteria can be used as an auxiliary diagnostic indicator to compensate for the subjective limitations of traditional symptom-based diagnosis; (2) Targeted Guidance for exploring disease mechanisms: This study found a potential association between gut microbiota imbalance and the occurrence and development of IBS in patients. Systematic analysis of the mechanisms provides a molecular basis for understanding the pathophysiology of IBS and emphasizes the potential value of gut microbiota as a biomarker in disease classification and individualized diagnosis and treatment.

### Research heterogeneity

(1) This study includes multiple IBS subtypes of IBS, with different types of IBS having different etiological mechanisms (Lee et al., 2021).The small sample size of the intestinal flora of patients with different types of IBS in this study, such as the studies by Xiaojun Zhuang et al. (Zhuang et al., 2018), and Siew Chien Ng et al. (Ng et al., 2013) may have contributed to the observed heterogeneity. The limited sample sizes may not adequately represent the population of patients with different types of IBS, thus compromising the reliability of the study results to some extent. (2) Due to the differences in the years of the included studies, the diagnostic criteria for IBS varied. At present, the diagnosis of IBS is mainly carried out through symptomatic manifestations and relevant examinations, and changes in diagnostic criteria in different periods may lead to differences in clinical manifestations and diagnostic results of the included cases, for example, Manning criteria, Rome I criteria, Rome II criteria, Rome III criteria, and Rome IV criteria, which is also one of the reasons for the heterogeneity. (3) Differences in microbiological detection methods likewise contribute to heterogeneity. Different detection methods may differ in sensitivity, specificity and detection range, and specific research methods include 16S *rRNA* sequencing and plate counting methods, which may affect the results of the detection of intestinal microorganisms. (4) The samples included in the study were of two types, stools and tissue. Different types of samples have different abundance of intestinal flora, which may affect the results of the assay and thus lead to heterogeneity. (5) Not all IBS patients included in the study underwent psychological assessment, including X. Huang et al. (Huang and Ding, 2023), Y. F. Jiang et al. (Jiang et al., 2006), J.-M. Si et al. (Si et al., 2004), X. Zhang et al. (Zhang et al., 2019), J. Zhao et al. (Zhao and Wang, 2021), X. Zhuang et al. (Zhuang et al., 2018), Y. Zhuang et al. (Zhuang et al., 2005), Lei Zhang et al. (Zhang et al., 2009), Siew Chien Ng et al. (Ng et al., 2013), Tingting Su et al. (Su et al., 2018). Mental health has been noted as one of the major etiological factors in IBS (Chaudhry, 2020; Omar et al., 2024). Psychological factors such as anxiety and depression may have an impact on intestinal function by affecting the intestinal nervous system and immune system, among other pathways. Therefore, the lack of comprehensive psychological assessment of IBS patients is one of the main reasons for heterogeneity.

## Advantages and current limitations

### Research advantage

(1) Geographical representativeness: This study was conducted on Chinese patients, with strong geographical specificity, which can provide targeted help and data support for the diagnosis and treatment of Chinese patients with IBS; (2) Bacterial flora richness: the inclusion of five types of intestinal flora for analysis provides valuable insights; (3) Scope and level of the study: the study is both comprehensive and well-structured. The overall changes in the intestinal flora of IBS patients were first comprehensively analyzed, and then categorized and examined, subdividing IBS into various types, and launching subgroup analysis of the IBS-D and IBS-C, so that the study is comprehensive and systematic.

### Research limitations

(1) Research methodology: There is a limited amount of research literature on intestinal flora and HC in Chinese IBS patients, and there is a lack of effective screening strategies for the selection of intestinal flora analysis techniques. Most of the included literature still relies on the culture medium counting method for quantitative analysis of intestinal flora, which is a traditional method with obvious limitations in resolution and precision; (2) Sample Level: For studies involving IBS-M and IBS-U, the sample size is relatively small. While an adequate sample size is crucial for ensuring the reliability and generalizability of research findings, the limited sample size in these subtypes may undermine the representativeness of the results. Future studies should consider expanding the sample size for constipation-predominant and mixed IBS cohorts to enhance the robustness and applicability of the findings. (3) While a core dysbiosis pattern transcends populations (e.g., pathobiont expansion-probiotic depletion), the directionality of taxon-specific changes (*Bacteroides*/*Enterococcus*), subtype manifestations (IBS-C), and underlying drivers (diet/antibiotics) exhibit significant geographic dependence. Our study provides a precise dysbiosis map for Chinese populations, and its divergences from multinational work underscore the necessity for standardized geographic stratification—future multi-center studies controlling for diet/medical practices must dissect universal patterns versus regional exceptions in IBS microbiota disruption.

### Recommendations for future research

Future studies on IBS should improve the scientific rigor and systematic approach in sample design: at the level of sample construction, it should cover a diverse group of patients with different subtypes (IBS-D, IBS-C, IBS-M, IBS-U), demographic characteristics (age, gender, and geographic location), and disease severity, so as to enhance the generalisability of the conclusions of the study by increasing the sample size; The diagnostic process should strictly follow Rome IV international standards, and the collection of biological samples (*e.g.*, stools or tissue) should be clearly regulated. At the technical level, high-throughput sequencing technologies (*e.g.*, 16S *rRNA* gene sequencing, metagenomic sequencing) should be prioritised to replace the traditional medium counting method to improve the accuracy and resolution of the intestinal flora analysis. In addition, psychological assessment tools (*e.g.*, HADS anxiety/depression scale, PHQ-9 depression scale) should be systematically integrated to quantitatively analyse the interaction between psychological state and intestinal flora dysregulation, so as to comprehensively reveal the pathological mechanism of IBS ‘gut-brain axis’.

## Conclusion

In summary, this systematic analysis demonstrated that, compared with healthy controls, the intestinal flora abundance of *Bifidobacterium* and *Lactobacillus* in Chinese IBS patients showed a decreasing trend, while at the same time, the flora abundance of *Enterobacter* and *Enterococcus* was significantly higher. No significant differences were found for the genus *Bacteroides*. More specifically, IBS-D patients exhibited increased abundances of *Enterobacter* and *Enterococcus*, along with decreased levels of putative beneficial bacteria such as *Lactobacillus*, *Bifidobacterium*, and *Bacteroides*. There was no significant difference in the number of *Enterobacter*, *Enterococcus*, *Lactobacillus*, and *Bifidobacterium* in Chinese IBS-C patients compared to HC. This result highlights a distinctive gut microbiota profile specific to Chinese IBS patients. Given the complexity of IBS pathogenesis and gut microbiota dysbiosis, future multidimensional studies must elucidate how geographic factors regulate gut flora-host interactions. This will provide a mechanistic basis for developing precision diagnostic and therapeutic strategies that account for geographic variation.

## PRISMA 2009 checklist statement

The authors have read the PRISMA 2009 Checklist, and the manuscript was prepared and revised according to the PRISMA 2009 Checklist.

## Data Availability

The original contributions presented in the study are included in the article/**Supplementary Material**. Further inquiries can be directed to the corresponding author.
