# Supplementary material for: Comparison with healthy controls: meta-analysis of changes in intestinal flora in Chinese patients with irritable bowel syndrome
Source: Front Cell Infect Microbiol. 2025 Oct 3;15:1657501. doi: 10.3389/fcimb.2025.1657501 (PMC12531269; doi:10.3389/fcimb.2025.1657501)
Supplement: Supplementary file 1 [file DataSheet1.docx]

Supplementary Material

**Supplementary Tables**

**Supplementary Table 1.** Datasheet for Figure 3-A.

|  | pinnual area $[\mathrm{mm}^{2}]$ | | | |
| --- | --- | --- | --- | --- |
| No. | *lancea* | outside | inside | *japonica* |
| 1 | 472.1172 | 403.003 | 761.778 | 721.1184 |
| 2 | 455.4567 | 375.837 | 759.171 | 983.7292 |
| 3 | 176.0926 | 382.04 | 915.658 | 668.5958 |
| 4 | 407.0018 | 478.949 | 1132.741 | 1316.715 |
| 5 | 375.1754 | 407.321 | 965.295 | 772.1445 |
| 6 | 308.6265 | 252.852 | 1023.337 | 925.0273 |
| 7 | 209.2729 | 336.275 | 882.561 | 1181.597 |
| 8 | 381.9693 | 333.879 | 1074.876 | 572.7334 |
| 9 | 650.3719 | 594.857 | 1368.503 | 516.4482 |
| 10 | 490.9746 | 362.124 | 950.647 | 1674.417 |
| 11 | 393.1808 | 993.413 | 617.987 | 1617.12 |
| 12 | 579.2183 | 671.612 | 717.215 | 1552.895 |
| 13 | 380.7108 | 884.906 | 589.3 | 938.3603 |
| 14 | 355.8056 | 1007.335 | 713.904 | 941.1831 |
| 15 | 380.0462 | 747.826 | 587.683 | 259.4224 |
| 16 |  | 684.985 | 565.718 | 1370.949 |
| 17 |  | 524.074 | 525.948 | 843.0967 |
| 18 |  | 440.481 | 771.649 | 723.0328 |
| 19 |  | 371.808 | 583.646 | 832.5128 |
| 20 |  | 342.232 | 642.897 | 461.5691 |
| 21 |  | 513.847 |  | 766.6151 |
| 22 |  | 466.7 |  | 480.7719 |
| 23 |  | 485.16 |  | 461.1618 |
| 24 |  |  |  | 701.6954 |

**Supplementary Table 2.** Datasheet for Figure 3-B.

|  | pinnual length$[\mathbf{mm}]$ | | | |
| --- | --- | --- | --- | --- |
| No. | *lancea* | outside | inside | *japonica* |
| 1 | 59.52 | 56.708 | 76.612 | 57.14667 |
| 2 | 57.91 | 53.953 | 70.557 | 71.43667 |
| 3 | 29.68 | 54.929 | 83.315 | 54.23333 |
| 4 | 59.25 | 60.006 | 90.578 | 81.9 |
| 5 | 55.1 | 52.454 | 89.506 | 56.77333 |
| 6 | 45.15 | 42.157 | 85.328 | 65.66333 |
| 7 | 29.45 | 50.68 | 78.489 | 78.49333 |
| 8 | 53.66 | 50.599 | 90.628 | 47.86 |
| 9 | 58.98 | 68.377 | 100.032 | 45.77 |
| 10 | 61.67 | 50.992 | 89.813 | 79.53 |
| 11 | 45.97 | 77.251 | 74.332 | 79.46667 |
| 12 | 60.17 | 70.599 | 69.705 | 88.82333 |
| 13 | 50.95 | 73.843 | 55.007 | 61.12333 |
| 14 | 46.18 | 84.04 | 73.958 | 58.44667 |
| 15 | 44.86 | 74.57 | 57.135 | 28.90667 |
| 16 |  | 61.867 | 58.104 | 88.78667 |
| 17 |  | 58.452 | 57.028 | 60.9 |
| 18 |  | 55.631 | 65.418 | 57.45333 |
| 19 |  | 48.331 | 60.018 | 53.17667 |
| 20 |  | 47.093 | 57.275 | 42.3 |
| 21 |  | 58.371 |  | 57.13667 |
| 22 |  | 54.266 |  | 43.93333 |
| 23 |  | 57.84 |  | 35.76667 |
| 24 |  |  |  | 54.10333 |

**Supplementary Table 3.** Datasheet for Figure 3-C.

|  | pinnual width $[\mathbf{mm}]$ | | | |
| --- | --- | --- | --- | --- |
| No. | *lancea* | outside | inside | *japonica* |
| 1 | 10.1 | 10.928 | 14.018 | 16.06667 |
| 2 | 10.01333 | 10.229 | 13.496 | 17.53333 |
| 3 | 7.553333 | 9.665 | 13.783 | 15.69667 |
| 4 | 8.746667 | 11.638 | 17.233 | 20.47 |
| 5 | 8.67 | 11.253 | 15.309 | 17.31667 |
| 6 | 8.703333 | 9.037 | 15.858 | 17.93667 |
| 7 | 9.046667 | 9.724 | 14.976 | 19.16667 |
| 8 | 9.063333 | 9.614 | 15.641 | 15.23667 |
| 9 | 14.04 | 12.613 | 19.635 | 14.36667 |
| 10 | 10.13667 | 10.271 | 15.305 | 26.80667 |
| 11 | 10.89 | 16.987 | 11.572 | 25.91 |
| 12 | 12.25667 | 15.181 | 15.142 | 22.26 |
| 13 | 9.513333 | 15.908 | 14.714 | 19.54667 |
| 14 | 9.81 | 16.712 | 13.435 | 20.50333 |
| 15 | 10.78667 | 14.871 | 14.141 | 11.42667 |
| 16 |  | 12.356 | 12.682 | 19.66 |
| 17 |  | 13.398 | 12.053 | 17.62667 |
| 18 |  | 10.863 | 16.114 | 16.02333 |
| 19 |  | 11.87 | 13.649 | 19.93333 |
| 20 |  | 10.345 | 14.885 | 13.89333 |
| 21 |  | 12.823 |  | 17.08333 |
| 22 |  | 12.393 |  | 13.93333 |
| 23 |  | 12.039 |  | 16.41667 |
| 24 |  |  |  | 16.51333 |

**Supplementary Table 4.** Datasheet for Figure 3-D.

|  | leaf index$[\mathbf{-}]$ | | | |
| --- | --- | --- | --- | --- |
| No. | *lancea* | outside | inside | *japonica* |
| 1 | 5.892739 | 5.189239 | 5.465259 | 3.556846 |
| 2 | 5.783622 | 5.274514 | 5.227993 | 4.074335 |
| 3 | 3.929832 | 5.68329 | 6.044765 | 3.455086 |
| 4 | 6.773628 | 5.156041 | 5.256078 | 4.000977 |
| 5 | 6.354864 | 4.661335 | 5.846626 | 3.278537 |
| 6 | 5.187668 | 4.664933 | 5.380754 | 3.660844 |
| 7 | 3.255711 | 5.211847 | 5.240986 | 4.095304 |
| 8 | 5.920559 | 5.263054 | 5.794259 | 3.141107 |
| 9 | 4.200855 | 5.421153 | 5.094576 | 3.185847 |
| 10 | 6.083854 | 4.964658 | 5.868213 | 2.966799 |
| 11 | 4.221304 | 4.547654 | 6.423436 | 3.067027 |
| 12 | 4.909165 | 4.650484 | 4.603421 | 3.990267 |
| 13 | 5.355992 | 4.641878 | 3.738412 | 3.127046 |
| 14 | 4.707441 | 5.028722 | 5.504875 | 2.850593 |
| 15 | 4.158838 | 5.014458 | 4.040379 | 2.529755 |
| 16 |  | 4.383682 | 4.581612 | 4.516107 |
| 17 |  | 4.362741 | 4.731436 | 3.454992 |
| 18 |  | 5.121145 | 4.0597 | 3.585604 |
| 19 |  | 4.071693 | 4.397245 | 2.667726 |
| 20 |  | 4.552247 | 3.847833 | 3.044626 |
| 21 |  | 4.552055 |  | 3.344585 |
| 22 |  | 4.378762 |  | 3.15311 |
| 23 |  | 4.804386 |  | 2.17868 |
| 24 |  |  |  | 3.276342 |

**Supplementary Table 5.** Datasheet for Figure 3-E.

|  | angle at the base $[\mathbf{℃}]$ | | | |
| --- | --- | --- | --- | --- |
| No. | *lancea* | outside | inside | *japonica* |
| 1 | 43.33333 | 33.949 | 96.628 | 150.3333 |
| 2 | 42.33333 | 36.477 | 84.26 | 150.6667 |
| 3 | 36.33333 | 63.616 | 87.679 | 143.3333 |
| 4 | 40.66667 | 46.633 | 86.947 | 159.6667 |
| 5 | 39.66667 | 42.869 | 83.787 | 167.6667 |
| 6 | 50.66667 | 40.731 | 84.425 | 148.6667 |
| 7 | 63 | 46.399 | 88.961 | 169.6667 |
| 8 | 39.66667 | 45.325 | 80.475 | 144.3333 |
| 9 | 54.66667 | 31.113 | 50.957 | 147.6667 |
| 10 | 40.33333 | 39.659 | 78.214 | 158.3333 |
| 11 | 64.33333 | 67.136 | 60.005 | 153 |
| 12 | 43.33333 | 41.756 | 68.611 | 166.3333 |
| 13 | 48 | 72.706 | 75.296 | 180 |
| 14 | 54.66667 | 66.153 | 74.663 | 144.3333 |
| 15 | 46.33333 | 51.052 | 68.316 | 127 |
| 16 |  | 59.082 | 81.431 | 180 |
| 17 |  | 42.153 | 83.838 | 167.3333 |
| 18 |  | 49.338 | 100.698 | 152.6667 |
| 19 |  | 43.562 | 93.755 | 148.3333 |
| 20 |  | 40.833 | 98.943 | 144.3333 |
| 21 |  | 38.889 |  | 157.6667 |
| 22 |  | 52.309 |  | 174.6667 |
| 23 |  | 47.683 |  | 180 |
| 24 |  |  |  | 169.6667 |

**Supplementary Table 6.** Datasheet for Figure 4.

|  | lamina area $[\mathrm{cm}^{2}]$ | | | |
| --- | --- | --- | --- | --- |
| No. | *lancea* | outside | inside | *japonica* |
| 1 | 292.746 | 261.2658 | 1096.641 | 1206.717 |
| 2 | 295.046 | 352.2494 | 895.2737 | 1688.681 |
| 3 | 314.696 | 180.6892 | 1628.626 | 1714.377 |
| 4 | 373.439 | 478.3885 | 1995.373 | 1157.202 |
| 5 | 372.142 | 260.7951 | 1536.249 | 909.145 |
| 6 | 352.96 | 234.0725 | 1714.193 | 1382.971 |
| 7 | 294.341 | 189.3632 | 1119.686 | 757.542 |
| 8 | 268.373 | 894.7943 | 1742.573 | 972.706 |
| 9 | 198.023 | 660.104 | 807.524 | 1030.9 |
| 10 | 175.817 | 1255.959 | 1124.631 | 784.912 |
| 11 | 145.077 | 753.1791 | 1155.863 | 1452.247 |
| 12 | 163.013 | 490.7988 | 668.1558 | 1135.371 |
| 13 | 149.839 | 419.9963 | 375.231 | 1688.125 |
| 14 | 170.921 | 145.452 | 965.3503 | 1982.694 |
| 15 | 210.746 | 205.8936 | 342.6301 | 1378.322 |
| 16 | 165.722 | 330.0214 | 615.5901 | 1305.289 |
| 17 | 245.603 | 437.1504 | 542.5804 | 1480.681 |
| 18 | 153.88 | 351.6016 | 499.593 | 809.383 |
| 19 | 167.689 |  | 463.9433 | 571.241 |
| 20 | 238.276 |  | 513.1048 | 1288.39 |

**Supplementary Table 7.** Datasheet for Figure 5-A.

|  | petiole length $[mm]$ | | | |
| --- | --- | --- | --- | --- |
| No. | *lancea* | outside | inside | *japonica* |
| 1 | 140.5 | 313 | 464 | 296 |
| 2 | 96 | 384 | 468 | 321 |
| 3 | 138 | 467 | 413 | 347 |
| 4 | 155 | 333 | 592 | 296 |
| 5 | 295 | 300 | 410 | 329 |
| 6 | 112 | 213 | 487 | 310 |
| 7 | 288 | 276 | 369 | 533 |
| 8 | 207 | 286 | 464 | 466 |
| 9 | 265 | 343 | 356 | 560 |
| 10 | 181 | 399 | 455 | 538 |
| 11 | 202 | 550 | 572 | 710 |
| 12 | 261 | 325 | 526 | 528 |
| 13 | 172 | 542 | 235 | 426 |
| 14 | 117 | 472 | 376 | 521 |
| 15 | 252 | 478 | 284 | 720 |
| 16 | 201 | 467 | 347 | 625 |
| 17 | 396 | 335 | 301 | 468 |
| 18 | 351 | 338 | 377 | 482 |
| 19 | 382 | 209 | 326 | 502 |
| 20 | 177 | 362 | 374 | 634 |
| 21 | 277 | 314 |  | 428 |
| 22 | 365 | 270 |  | 425 |
| 23 | 234 | 246 |  | 565 |
| 24 | 266 |  |  | 446 |
| 25 | 285 |  |  | 635 |
| 26 | 300 |  |  | 543 |
| 27 | 296 |  |  | 501 |
| 28 | 242 |  |  | 415 |
| 29 | 366 |  |  | 367 |
| 30 | 277 |  |  | 391 |
| 31 | 281 |  |  | 408 |
| 32 | 272 |  |  | 522 |
| 33 | 144 |  |  | 420 |
| 34 | 95 |  |  | 672 |
| 35 | 139 |  |  | 483 |
| 36 | 183 |  |  | 563 |
| 37 | 134 |  |  | 566 |
| 38 | 78 |  |  | 595 |
| 39 | 201 |  |  | 488 |
| 40 | 112 |  |  | 490 |
| 41 | 169 |  |  | 437 |
| 42 | 86 |  |  | 396 |
| 43 | 158 |  |  | 499 |
| 44 | 195 |  |  | 770 |
| 45 | 234 |  |  | 723 |
| 46 | 268 |  |  | 586 |
| 47 | 224 |  |  | 614 |
| 48 | 273 |  |  | 587 |
| 49 | 353 |  |  | 534 |
| 50 | 329 |  |  | 702 |
| 51 | 298 |  |  | 526 |
| 52 | 313 |  |  | 657 |
| 53 | 370 |  |  | 536 |
| 54 | 125 |  |  | 468 |
| 55 | 232 |  |  | 421 |
| 56 | 220 |  |  | 470 |
| 57 | 334 |  |  | 448 |
| 58 | 257 |  |  | 596 |

**Supplementary Table 8.** Datasheet for Figure 5-B.

|  | cross-sectional area $[\mathrm{mm}^{2}]$ | | | |
| --- | --- | --- | --- | --- |
| No. | *lancea* | outside | inside | *japonica* |
| 1 | 8.635924 | 28.58747 | 41.37194 | 9.439858 |
| 2 | 15.58261 | 34.16199 | 37.18291 | 15.11734 |
| 3 | 8.483714 | 41.2982 | 60.25655 | 17.33782 |
| 4 | 11.01812 | 34.14621 | 78.3375 | 10.62534 |
| 5 | 21.31178 | 29.76266 | 59.61995 | 15.29956 |
| 6 | 11.83249 | 16.65641 | 79.44899 | 12.49074 |
| 7 | 13.34596 | 27.9586 | 36.83802 | 43.41445 |
| 8 | 23.97161 | 20.46543 | 70.42934 | 20.95835 |
| 9 | 25.24717 | 35.56911 | 32.53535 | 30.27199 |
| 10 | 19.19332 | 40.0107 | 60.99636 | 35.9732 |
| 11 | 13.61692 | 39.10356 | 51.2133 | 44.84788 |
| 12 | 15.53533 | 34.81136 | 34.72298 | 42.07409 |
| 13 | 17.20964 | 41.54631 | 12.77888 | 23.54184 |
| 14 | 17.90684 | 41.16837 | 37.10289 | 33.25062 |
| 15 | 13.99485 | 34.89634 | 15.51729 | 70.91706 |
| 16 | 16.2773 | 36.11952 | 25.12559 | 44.83815 |
| 17 | 31.84821 | 25.80567 | 24.8856 | 28.16312 |
| 18 | 17.963 | 24.52257 | 23.66469 | 43.05898 |
| 19 | 23.48678 | 18.56084 | 22.73693 | 32.08312 |
| 20 | 16.62531 | 17.21742 | 26.94853 | 49.03908 |
| 21 | 21.68712 | 16.32843 |  | 23.84987 |
| 22 | 28.25077 | 20.84871 |  | 20.55882 |
| 23 | 16.89148 | 21.06901 |  | 34.21312 |
| 24 | 16.66764 |  |  | 29.49673 |
| 25 | 20.21474 |  |  | 46.11544 |
| 26 | 22.88336 |  |  | 39.02447 |
| 27 | 26.67259 |  |  | 33.72123 |
| 28 | 22.11493 |  |  | 31.03721 |
| 29 | 24.04402 |  |  | 26.09344 |
| 30 | 22.98845 |  |  | 19.5605 |
| 31 | 16.49957 |  |  | 24.15335 |
| 32 | 8.685875 |  |  | 34.9942 |
| 33 | 14.36517 |  |  | 27.42893 |
| 34 | 6.861238 |  |  | 65.14862 |
| 35 | 10.1907 |  |  | 31.83454 |
| 36 | 10.32249 |  |  | 51.21943 |
| 37 | 8.037765 |  |  | 61.58778 |
| 38 | 15.38909 |  |  | 58.30168 |
| 39 | 16.99696 |  |  | 39.61352 |
| 40 | 13.60498 |  |  | 37.18075 |
| 41 | 14.6681 |  |  | 26.9452 |
| 42 | 11.08197 |  |  | 18.64504 |
| 43 | 10.99212 |  |  | 31.67762 |
| 44 | 9.297072 |  |  | 63.2434 |
| 45 | 17.13605 |  |  | 52.76556 |
| 46 | 25.2961 |  |  | 26.21031 |
| 47 | 12.98687 |  |  | 36.56107 |
| 48 | 14.87301 |  |  | 37.03514 |
| 49 | 27.3017 |  |  | 36.50546 |
| 50 | 21.78734 |  |  | 45.49419 |
| 51 | 15.88421 |  |  | 23.15464 |
| 52 | 16.72309 |  |  | 48.15418 |
| 53 | 26.26607 |  |  | 29.84741 |
| 54 | 10.06236 |  |  | 29.08109 |
| 55 | 17.03591 |  |  | 21.34453 |
| 56 | 13.6031 |  |  | 26.7915 |
| 57 | 16.97379 |  |  | 28.31109 |
| 58 | 17.94148 |  |  | 49.92808 |

**Supplementary Table 9.** Datasheet for Figure 8-A.

|  | bending modulus $[\mathrm{MPa}]$ | | | |
| --- | --- | --- | --- | --- |
| No. | *lancea* | outside | inside | *japonica* |
| 1 | 604.21 | 283.07 | 303.66 | 893 |
| 2 | 755.33 | 369.97 | 573.39 | 807.18 |
| 3 | 534.47 | 254.98 | 1322.4 | 692.2 |
| 4 | 494.46 | 414.75 | 1147.5 | 375.2 |
| 5 | 387.56 | 229.26 | 1102.9 | 384.92 |
| 6 | 617.87 | 301.94 | 1216.3 | 741.79 |
| 7 | 549.45 | 442.35 | 956.16 | 660.4 |
| 8 | 799.94 | 217.94 | 1150 | 723.61 |
| 9 | 466.37 | 277.79 | 793.73 | 921.52 |
| 10 | 435.25 | 238.47 | 768.42 | 497.12 |
| 11 | 654.33 | 443.64 | 1559 | 468.34 |
| 12 | 571.28 | 397.23 | 962.25 | 395.67 |
| 13 | 626.21 | 423.71 | 658.05 | 697.93 |
| 14 | 772.39 | 578.52 | 897.17 | 857.07 |
| 15 | 419.15 | 453.45 | 665.41 | 139.57 |
| 16 | 229.21 | 323.3 | 853.2 | 487.99 |
| 17 | 547.29 | 342.05 | 1041.3 | 457.23 |
| 18 | 318.25 | 339.92 | 941.5 | 386.05 |
| 19 | 451.77 | 516.67 | 580.8 | 657.07 |
| 20 | 667.97 | 223.96 | 682.01 | 1022.4 |
| 21 | 638.21 | 289.91 |  | 675.51 |
| 22 | 853.24 | 296.5 |  | 772.23 |
| 23 | 979.49 | 210.63 |  | 944.27 |
| 24 | 719.6 |  |  | 782.23 |
| 25 | 693.29 |  |  | 736.58 |
| 26 | 635.07 |  |  | 654.09 |
| 27 | 899.73 |  |  | 900.82 |
| 28 | 659.68 |  |  | 679.76 |
| 29 | 730.96 |  |  | 648.17 |
| 30 | 760.91 |  |  | 777.08 |
| 31 | 706.52 |  |  | 798.26 |
| 32 | 576.37 |  |  | 710.16 |
| 33 | 593.81 |  |  | 502.38 |
| 34 | 775.01 |  |  | 689.63 |
| 35 | 199.81 |  |  | 430.9 |
| 36 | 490.75 |  |  | 871.4 |
| 37 | 487.1 |  |  | 520.42 |
| 38 | 433.65 |  |  | 711.63 |
| 39 | 569.34 |  |  | 537.43 |
| 40 | 581.78 |  |  | 858.13 |
| 41 | 621.32 |  |  | 855.82 |
| 42 | 525.64 |  |  | 801.56 |
| 43 | 557.09 |  |  | 752.53 |
| 44 | 735.59 |  |  | 821.15 |
| 45 | 383.48 |  |  | 1166.9 |
| 46 | 735.19 |  |  | 711.24 |
| 47 | 709.07 |  |  | 763.42 |
| 48 | 791.35 |  |  | 493.33 |
| 49 | 485.69 |  |  | 685.35 |
| 50 | 479.32 |  |  | 821.55 |
| 51 | 474.68 |  |  | 652.34 |
| 52 | 899.37 |  |  | 705.98 |
| 53 | 651.12 |  |  | 727.37 |
| 54 | 576.58 |  |  | 982.56 |
| 55 | 702.43 |  |  | 948.58 |
| 56 | 326.02 |  |  | 586.87 |
| 57 | 813.93 |  |  | 262.8 |
| 58 | 595.41 |  |  | 310.53 |
| 59 | 696.78 |  |  | 581.42 |
| 60 | 495.62 |  |  | 503.56 |
| 61 | 480.18 |  |  | 387.55 |
| 62 | 631.68 |  |  | 577.53 |
| 63 | 584.04 |  |  | 724.15 |
| 64 | 500.26 |  |  | 756.52 |
| 65 | 547.09 |  |  | 753.15 |
| 66 | 511.3 |  |  | 440.8 |
| 67 | 744.04 |  |  | 902.54 |
| 68 | 355.86 |  |  | 287.14 |
| 69 | 421.4 |  |  | 906.42 |
| 70 | 615.49 |  |  | 766.61 |
| 71 | 350.72 |  |  | 490.44 |
| 72 | 627.45 |  |  | 682.34 |
| 73 | 676.48 |  |  | 587.75 |
| 74 | 440.25 |  |  | 323.37 |

**Supplementary Table 10.** Datasheet for Figure 8-B.

|  | bending strength $[{N/mm}^{2}]$ | | | |
| --- | --- | --- | --- | --- |
| No. | *lancea* | outside | inside | *japonica* |
| 1 | 12.35391 | 8.52889 | 13.88093 | 10.5999 |
| 2 | 12.5384 | 7.901796 | 8.165123 | 12.80896 |
| 3 | 10.72718 | 5.892334 | 16.06122 | 13.7918 |
| 4 | 9.228942 | 8.326308 | 11.2562 | 8.172389 |
| 5 | 9.002413 | 4.207204 | 13.33519 | 9.555426 |
| 6 | 9.479987 | 7.850035 | 12.26209 | 9.902124 |
| 7 | 9.755992 | 11.7694 | 13.21314 | 11.18571 |
| 8 | 12.34724 | 8.500582 | 11.99832 | 9.668366 |
| 9 | 10.08254 | 6.118709 | 11.57193 | 14.09615 |
| 10 | 7.964821 | 8.192163 | 10.42352 | 9.353012 |
| 11 | 10.76855 | 6.85002 | 15.20213 | 4.767804 |
| 12 | 12.73674 | 10.98999 | 15.05247 | 5.953648 |
| 13 | 12.35183 | 6.866593 | 23.17543 | 13.14485 |
| 14 | 12.81701 | 8.49782 | 12.40131 | 11.83088 |
| 15 | 10.30361 | 6.223452 | 22.1176 | 10.88335 |
| 16 | 6.179624 | 6.158757 | 17.46625 | 6.28715 |
| 17 | 9.247193 | 7.926416 | 15.4434 | 4.934811 |
| 18 | 5.509977 | 6.739754 | 18.28042 | 4.907406 |
| 19 | 6.956441 | 8.834959 | 21.17661 | 7.594221 |
| 20 | 10.63605 | 6.900366 | 16.52709 | 11.34012 |
| 21 | 11.94514 | 8.407458 |  | 10.06266 |
| 22 | 13.41741 | 7.885679 |  | 10.4884 |
| 23 | 12.80109 | 11.29408 |  | 11.19927 |
| 24 | 15.46175 |  |  | 10.28055 |
| 25 | 15.37759 |  |  | 10.19633 |
| 26 | 11.95652 |  |  | 9.651702 |
| 27 | 13.53534 |  |  | 11.8923 |
| 28 | 11.83988 |  |  | 10.01998 |
| 29 | 13.94184 |  |  | 9.560627 |
| 30 | 10.10361 |  |  | 8.532785 |
| 31 | 12.19947 |  |  | 10.11242 |
| 32 | 11.33178 |  |  | 9.175827 |
| 33 | 10.64061 |  |  | 9.520084 |
| 34 | 11.55113 |  |  | 10.61031 |
| 35 | 5.654121 |  |  | 9.762238 |
| 36 | 10.20811 |  |  | 11.39653 |
| 37 | 12.92264 |  |  | 8.515492 |
| 38 | 10.67541 |  |  | 9.06396 |
| 39 | 13.82388 |  |  | 9.371639 |
| 40 | 12.78167 |  |  | 8.581345 |
| 41 | 11.54346 |  |  | 9.782623 |
| 42 | 12.57756 |  |  | 10.20707 |
| 43 | 12.86293 |  |  | 9.135236 |
| 44 | 15.86346 |  |  | 9.526147 |
| 45 | 10.71377 |  |  | 10.97585 |
| 46 | 12.19318 |  |  | 8.038406 |
| 47 | 13.7686 |  |  | 10.37313 |
| 48 | 10.76099 |  |  | 7.486537 |
| 49 | 9.14224 |  |  | 8.777336 |
| 50 | 14.35423 |  |  | 10.27617 |
| 51 | 12.23673 |  |  | 8.875745 |
| 52 | 11.0326 |  |  | 9.073597 |
| 53 | 13.67428 |  |  | 7.692333 |
| 54 | 7.620154 |  |  | 8.779303 |
| 55 | 11.07343 |  |  | 10.0443 |
| 56 | 10.7959 |  |  | 9.45794 |
| 57 | 9.007211 |  |  | 8.330053 |
| 58 | 6.764169 |  |  | 5.80922 |
| 59 | 7.638731 |  |  | 6.691265 |
| 60 | 11.61842 |  |  | 8.566049 |
| 61 | 10.72864 |  |  | 7.451657 |
| 62 | 8.644405 |  |  | 8.567764 |
| 63 | 11.61183 |  |  | 7.143936 |
| 64 | 8.919938 |  |  | 9.487931 |
| 65 | 13.43395 |  |  | 9.125118 |
| 66 | 8.702265 |  |  | 5.528625 |
| 67 | 6.911324 |  |  | 7.808991 |
| 68 | 10.63755 |  |  | 3.752225 |
| 69 | 13.31464 |  |  | 7.083025 |
| 70 | 12.83873 |  |  | 6.507596 |
| 71 | 11.52066 |  |  | 7.038893 |
| 72 | 8.821271 |  |  | 6.471294 |
| 73 |  |  |  | 5.448692 |
| 74 |  |  |  | 4.947944 |

**Supplementary Table 11.** Datasheet for Figure 8-C.

|  | breaking strain $[\%]$ | | | |
| --- | --- | --- | --- | --- |
| No. | *lancea* | outside | inside | *japonica* |
| 1 | 7.348488 | 9.556602 | 3.029091 | 4.160188 |
| 2 | 10.51563 | 9.33352 | 4.935428 | 4.076471 |
| 3 | 11.22041 | 7.103045 | 2.939584 | 4.796828 |
| 4 | 7.987742 | 10.34616 | 1.785289 | 4.233388 |
| 5 | 13.66181 | 8.155335 | 3.062536 | 5.000815 |
| 6 | 13.08557 | 12.40259 | 2.019872 | 3.763829 |
| 7 | 5.889483 | 9.106826 | 3.06534 | 6.123003 |
| 8 | 11.28454 | 9.639528 | 2.014675 | 7.58479 |
| 9 | 7.73383 | 9.200076 | 4.076554 | 3.607322 |
| 10 | 13.41975 | 7.273168 | 3.040846 | 3.37854 |
| 11 | 9.237208 | 5.323775 | 2.478131 | 5.637725 |
| 12 | 11.87762 | 6.93874 | 2.984717 | 7.784444 |
| 13 | 9.130958 | 2.897825 | 5.45054 | 8.830024 |
| 14 | 11.87772 | 3.116786 | 2.590626 | 10.09564 |
| 15 | 12.02167 | 3.457657 | 5.753601 | 12.13205 |
| 16 | 14.89309 | 3.825008 | 5.336756 | 5.539276 |
| 17 | 13.39695 | 11.73368 | 3.804406 | 5.211779 |
| 18 | 15.83326 | 12.07063 | 4.187303 | 4.773664 |
| 19 | 13.73249 | 13.92986 | 4.298382 | 3.111966 |
| 20 | 11.21746 | 13.40179 | 4.707393 | 3.805002 |
| 21 | 10.08011 | 16.00026 |  | 2.865615 |
| 22 | 6.57079 | 14.50048 |  | 3.956667 |
| 23 | 9.403543 | 13.36681 |  | 2.899849 |
| 24 | 7.300977 |  |  | 4.110927 |
| 25 | 6.855123 |  |  | 4.299694 |
| 26 | 9.239969 |  |  | 6.930808 |
| 27 | 7.885472 |  |  | 3.109682 |
| 28 | 8.148785 |  |  | 3.052051 |
| 29 | 14.51393 |  |  | 2.840613 |
| 30 | 8.078671 |  |  | 3.788963 |
| 31 | 7.521885 |  |  | 3.00559 |
| 32 | 10.61679 |  |  | 3.410049 |
| 33 | 12.90286 |  |  | 2.931419 |
| 34 | 12.65059 |  |  | 2.19133 |
| 35 | 16.71875 |  |  | 4.536027 |
| 36 | 10.77107 |  |  | 3.672935 |
| 37 | 9.145964 |  |  | 7.001579 |
| 38 | 11.89715 |  |  | 4.076014 |
| 39 | 12.81702 |  |  | 4.091434 |
| 40 | 14.40787 |  |  | 2.390777 |
| 41 | 13.90251 |  |  | 2.174472 |
| 42 | 9.25005 |  |  | 2.883723 |
| 43 | 8.19588 |  |  | 2.773945 |
| 44 | 11.48208 |  |  | 3.403758 |
| 45 | 8.326154 |  |  | 2.670807 |
| 46 | 9.278066 |  |  | 2.512498 |
| 47 | 7.492663 |  |  | 3.789351 |
| 48 | 11.03257 |  |  | 2.242425 |
| 49 | 9.416719 |  |  | 2.911672 |
| 50 | 10.17043 |  |  | 3.079577 |
| 51 | 10.82066 |  |  | 4.234823 |
| 52 | 10.86815 |  |  | 3.300146 |
| 53 | 8.312733 |  |  | 3.275044 |
| 54 | 12.00805 |  |  | 2.289241 |
| 55 | 12.01425 |  |  | 2.852897 |
| 56 | 10.42577 |  |  | 2.740009 |
| 57 | 10.71332 |  |  | 2.359757 |
| 58 | 10.96359 |  |  | 5.194675 |
| 59 | 14.86561 |  |  | 2.972697 |
| 60 | 10.7728 |  |  | 6.815142 |
| 61 | 10.48235 |  |  | 7.499214 |
| 62 | 10.72051 |  |  | 1.961606 |
| 63 | 12.90843 |  |  | 3.551957 |
| 64 | 9.550078 |  |  | 3.033223 |
| 65 | 14.79322 |  |  | 2.473012 |
| 66 | 13.68461 |  |  | 3.600303 |
| 67 | 11.93609 |  |  | 2.640663 |
| 68 | 13.4769 |  |  | 8.301227 |
| 69 | 17.06231 |  |  | 2.251808 |
| 70 | 19.19865 |  |  | 3.480185 |
| 71 | 14.40016 |  |  | 1.960406 |
| 72 | 13.74897 |  |  | 3.802592 |
| 73 |  |  |  | 4.310537 |
| 74 |  |  |  | 4.352579 |

**Supplementary Table 12.** Datasheet for Figure 9.

|  | weight per unit volume in a petiole $[{mg/mm}^{2}]$ | | | |
| --- | --- | --- | --- | --- |
| No. | *lancea* | outside | inside | *japonica* |
| 1 | 0.182609 | 0.165286 | 0.215881 | 0.16485 |
| 2 | 0.205382 | 0.175616 | 0.199363 | 0.166711 |
| 3 | 0.231371 | 0.149043 | 0.210576 | 0.13786 |
| 4 | 0.165672 | 0.161838 | 0.205926 | 0.156613 |
| 5 | 0.202792 | 0.174269 | 0.207201 | 0.152057 |
| 6 | 0.180923 | 0.174919 | 0.194347 | 0.183282 |
| 7 | 0.245103 | 0.203344 | 0.202785 | 0.203816 |
| 8 | 0.241133 | 0.150743 | 0.196121 | 0.2294 |
| 9 | 0.172734 | 0.142039 | 0.213846 | 0.156024 |
| 10 | 0.155948 | 0.172501 | 0.192127 | 0.22044 |
| 11 | 0.257758 | 0.16155 | 0.176337 | 0.209619 |
| 12 | 0.181445 | 0.155506 | 0.18738 | 0.161645 |
| 13 | 0.151494 | 0.156169 | 0.300664 | 0.144258 |
| 14 | 0.173401 | 0.176354 | 0.186658 | 0.177437 |
| 15 | 0.107175 | 0.170496 | 0.283633 | 0.220815 |
| 16 | 0.149821 | 0.170853 | 0.267466 | 0.235935 |
| 17 | 0.163172 | 0.147128 | 0.249224 | 0.214193 |
| 18 | 0.166016 | 0.141153 | 0.275298 | 0.144198 |
| 19 | 0.205447 | 0.142836 | 0.32585 | 0.166131 |
| 20 | 0.247069 | 0.134525 | 0.246584 | 0.121295 |
| 21 | 0.133539 | 0.171747 |  | 0.124906 |
| 22 | 0.138271 | 0.144016 |  | 0.122049 |
| 23 | 0.264588 | 0.167319 |  | 0.141865 |
| 24 | 0.226854 |  |  | 0.151817 |
| 25 | 0.196731 |  |  | 0.147929 |
| 26 | 0.197731 |  |  | 0.172485 |
| 27 | 0.228867 |  |  | 0.155774 |
| 28 | 0.178022 |  |  | 0.193825 |
| 29 | 0.207653 |  |  | 0.164063 |
| 30 | 0.214743 |  |  | 0.127343 |
| 31 | 0.186411 |  |  | 0.191892 |
| 32 | 0.17617 |  |  | 0.142128 |
| 33 | 0.244588 |  |  | 0.136198 |
| 34 | 0.254617 |  |  | 0.133003 |
| 35 | 0.159644 |  |  | 0.14577 |
| 36 | 0.19751 |  |  | 0.12999 |
| 37 | 0.199211 |  |  | 0.144808 |
| 38 | 0.183992 |  |  | 0.107621 |
| 39 | 0.209953 |  |  | 0.123259 |
| 40 | 0.264279 |  |  | 0.142112 |
| 41 | 0.199309 |  |  | 0.134401 |
| 42 | 0.188734 |  |  | 0.105882 |
| 43 | 0.186022 |  |  | 0.163763 |
| 44 | 0.197722 |  |  | 0.153264 |
| 45 | 0.224977 |  |  | 0.14786 |
| 46 | 0.221226 |  |  | 0.177443 |
| 47 | 0.20103 |  |  | 0.179134 |
| 48 | 0.230482 |  |  | 0.170517 |
| 49 | 0.206712 |  |  | 0.174107 |
| 50 | 0.215918 |  |  | 0.192443 |
| 51 | 0.189474 |  |  | 0.216474 |
| 52 | 0.267757 |  |  | 0.156729 |
| 53 | 0.233349 |  |  | 0.164309 |
| 54 | 0.202252 |  |  | 0.170808 |
| 55 | 0.234203 |  |  | 0.1468 |
| 56 | 0.245337 |  |  | 0.143978 |
| 57 | 0.180172 |  |  | 0.152877 |
| 58 | 0.125052 |  |  | 0.195227 |
| 59 | 0.210599 |  |  | 0.174429 |
| 60 | 0.210428 |  |  | 0.166577 |
| 61 | 0.239074 |  |  | 0.192354 |
| 62 | 0.216063 |  |  | 0.155173 |
| 63 | 0.223411 |  |  | 0.175156 |
| 64 | 0.190198 |  |  | 0.155998 |
| 65 | 0.198413 |  |  | 0.171885 |
| 66 | 0.216181 |  |  | 0.16093 |
| 67 | 0.224448 |  |  | 0.161586 |
| 68 | 0.2513 |  |  | 0.172044 |
| 69 | 0.182324 |  |  | 0.169663 |
| 70 | 0.199922 |  |  | 0.177889 |
| 71 | 0.199262 |  |  | 0.172617 |
| 72 | 0.211675 |  |  | 0.165398 |
| 73 | 0.188078 |  |  | 0.162193 |
| 74 | 0.191384 |  |  | 0.177279 |
| 75 | 0.275993 |  |  | 0.158228 |
| 76 | 0.224731 |  |  | 0.170875 |
| 77 | 0.210584 |  |  | 0.16199 |
| 78 | 0.191904 |  |  | 0.141675 |
| 79 | 0.210421 |  |  | 0.158851 |
| 80 | 0.136036 |  |  | 0.169483 |
| 81 | 0.124277 |  |  | 0.19439 |
| 82 | 0.129038 |  |  | 0.135367 |
| 83 | 0.134091 |  |  | 0.141057 |
| 84 | 0.152997 |  |  | 0.157705 |
| 85 | 0.173117 |  |  | 0.167659 |
| 86 | 0.14192 |  |  | 0.12068 |
| 87 | 0.155211 |  |  | 0.11755 |
| 88 | 0.172717 |  |  | 0.150511 |
| 89 | 0.175332 |  |  | 0.111175 |
| 90 | 0.152307 |  |  | 0.15893 |
| 91 | 0.151068 |  |  | 0.139249 |
| 92 | 0.171201 |  |  | 0.165419 |
| 93 | 0.164766 |  |  | 0.122066 |
| 94 | 0.161329 |  |  | 0.149492 |
| 95 | 0.174876 |  |  | 0.116818 |
| 96 | 0.164058 |  |  | 0.109214 |
| 97 | 0.174431 |  |  | 0.153952 |
| 98 | 0.180873 |  |  | 0.1232 |
| 99 | 0.198773 |  |  | 0.156795 |
| 100 | 0.154703 |  |  | 0.141573 |
| 101 | 0.154348 |  |  | 0.15117 |
| 102 | 0.157969 |  |  |  |
| 103 | 0.168762 |  |  |  |
| 104 | 0.14999 |  |  |  |
| 105 | 0.150273 |  |  |  |
| 106 | 0.135766 |  |  |  |
| 107 | 0.139557 |  |  |  |
| 108 | 0.209988 |  |  |  |
| 109 | 0.168202 |  |  |  |
| 110 | 0.168116 |  |  |  |
| 111 | 0.195164 |  |  |  |

**Supplementary Table 13.** Datasheet for Figure 10-D.

|  | the cross-sectional area ratio of sterome $[\boldsymbol{\%}]$ | | | |
| --- | --- | --- | --- | --- |
| No. | *lancea* | outside | inside | *japonica* |
| 1 | 18.1584 | 22.40437 | 17.25576 | 17.00552 |
| 2 | 19.44385 | 17.86114 | 17.90136 | 12.30938 |
| 3 | 18.47061 | 14.63508 | 16.59944 | 11.64477 |
| 4 | 21.59515 | 17.86422 | 17.50744 | 10.59554 |
| 5 | 16.93228 | 15.39781 | 18.67619 | 15.3722 |
| 6 | 15.88581 | 18.83449 | 16.27301 | 14.45767 |
| 7 | 15.49398 | 17.55858 | 15.66436 | 14.63452 |
| 8 | 17.00552 | 19.63237 | 17.09652 | 14.37255 |
| 9 | 18.32487 | 16.30876 | 17.48973 | 18.19314 |
| 10 | 16.13782 | 15.67987 | 15.50463 | 13.90918 |
| 11 | 16.93228 | 16.15395 | 16.38105 | 15.81748 |
| 12 | 15.95543 | 14.51615 | 15.98116 | 23.41866 |
| 13 | 23.11187 | 15.63106 | 23.96129 | 18.40103 |
| 14 | 16.18762 | 16.12649 | 16.83051 | 17.30335 |
| 15 | 19.6537 | 19.03509 | 22.20425 | 16.02121 |
| 16 | 22.96766 | 16.87321 | 16.47715 | 15.81921 |
| 17 | 20.54351 | 15.84551 | 19.40583 |  |
| 18 |  | 16.30757 | 19.73956 |  |
| 19 |  | 19.88831 | 21.27195 |  |
| 20 |  | 18.31749 | 19.41893 |  |
| 21 |  | 20.20211 |  |  |
| 22 |  | 16.79128 |  |  |
| 23 |  | 19.96946 |  |  |

**Supplementary Table 14.** Datasheet for Figure 10-E.

|  | the cell wall area ratio of the sterome $[\boldsymbol{\%}]$ | | | |
| --- | --- | --- | --- | --- |
| No. | *lancea* | outside | inside | *japonica* |
| 1 | 44.97368 | 54.44584 | 75.92348 | 65.4375 |
| 2 | 48.59753 | 65.63453 | 68.69915 | 62.25957 |
| 3 | 51.8051 | 53.27265 | 73.60727 | 64.10058 |
| 4 | 47.13272 | 54.55808 | 75.90521 | 59.02597 |
| 5 | 46.63646 | 65.26301 | 78.63836 | 58.52221 |
| 6 | 41.11986 | 59.24616 | 69.81573 | 60.88532 |
| 7 | 56.61446 | 70.05725 | 70.4226 | 47.30346 |
| 8 | 63.35756 | 60.10812 | 65.93009 | 60.04149 |
| 9 | 58.06294 | 58.90643 | 71.25519 | 66.18952 |
| 10 | 47.33836 | 65.06039 | 75.17544 | 49.85836 |
| 11 | 44.57773 | 60.22489 | 68.60703 | 53.04727 |
| 12 | 37.93128 | 61.86408 | 74.38493 | 62.85496 |
| 13 | 50.73524 | 59.00388 | 75.97913 | 72.61067 |
| 14 | 63.37789 | 66.69047 | 66.27683 | 67.58933 |
| 15 | 59.19545 | 61.78076 | 71.367 | 69.00115 |
| 16 | 58.75684 | 64.71031 | 76.66691 | 57.13206 |
| 17 | 57.13529 | 54.06813 | 79.50315 | 66.03533 |
| 18 | 61.06335 | 55.03856 | 80.33987 | 62.406 |
| 19 | 55.71569 | 52.16303 | 76.81716 | 62.61367 |
| 20 | 57.92481 | 53.25241 | 81.16388 | 70.30712 |
| 21 | 34.01768 | 59.8926 |  | 60.442 |
| 22 | 60.22858 | 55.37872 |  | 63.23667 |
| 23 | 47.71257 | 54.83628 |  | 70.24233 |
| 24 | 56.88795 |  |  | 62.66233 |
| 25 | 53.79971 |  |  | 46.61533 |
| 26 | 36.64017 |  |  | 54.903 |
| 27 | 52.27742 |  |  | 62.52367 |
| 28 | 55.74607 |  |  | 57.77667 |
| 29 | 47.97429 |  |  | 57.39933 |
| 30 | 25.14336 |  |  | 49.21267 |
| 31 | 47.32083 |  |  | 51.40633 |
| 32 | 56.96963 |  |  | 55.19067 |
| 33 | 60.45895 |  |  | 53.34767 |
| 34 | 48.207 |  |  | 52.79867 |
| 35 | 59.39575 |  |  | 59.59067 |
| 36 | 26.41014 |  |  | 60.17967 |
| 37 | 50.80424 |  |  | 53.12267 |
| 38 | 47.56765 |  |  | 68.44433 |
| 39 | 49.20895 |  |  | 65.42667 |
| 40 | 41.38886 |  |  | 60.494 |
| 41 | 50.68255 |  |  | 64.016 |
| 42 | 56.9807 |  |  | 59.55967 |
| 43 | 61.25414 |  |  | 46.72483 |
| 44 | 50.30982 |  |  | 58.70401 |
| 45 | 39.11911 |  |  | 57.86086 |
| 46 | 52.33752 |  |  | 57.94154 |
| 47 | 51.28599 |  |  | 58.06256 |
| 48 | 56.50361 |  |  | 59.16843 |
| 49 | 52.49455 |  |  | 53.179 |
| 50 | 53.55307 |  |  | 39.40917 |
| 51 | 45.81867 |  |  | 47.38995 |
| 52 | 59.05207 |  |  | 46.30242 |
| 53 | 61.73689 |  |  | 58.60699 |
| 54 | 68.5298 |  |  | 51.71841 |
| 55 | 73.34201 |  |  | 63.54422 |
| 56 | 60.37365 |  |  | 61.33615 |
| 57 | 64.21201 |  |  | 58.96139 |
| 58 | 62.70474 |  |  | 70.78354 |
| 59 | 63.05313 |  |  | 68.02256 |
| 60 | 69.33594 |  |  | 66.32273 |
| 61 |  |  |  | 67.10487 |
| 62 |  |  |  | 59.29578 |
| 63 |  |  |  | 63.9443 |

**Supplementary Table 15.** Datasheet for Figure 10-F.

|  | the length of sclerenchyma cells $[\boldsymbol{\mu m}]$ | | | |
| --- | --- | --- | --- | --- |
| No. | *lancea* | outside | inside | *japonica* |
| 1 | 529.1692 | 679.6012 | 763.7734 | 707.2205 |
| 2 | 719.5069 | 673.4324 | 805.1352 | 707.1112 |
| 3 | 557.5448 | 708.1289 | 734.4735 | 934.6882 |
| 4 | 685.4506 | 670.8008 | 707.8077 | 793.3934 |
| 5 | 646.9329 | 425.6538 | 687.262 | 897.2289 |
| 6 | 583.7528 | 398.6335 | 792.5979 | 687.505 |
| 7 | 659.8826 | 416.5558 | 636.1257 | 772.6754 |
| 8 | 625.6274 | 662.3985 | 739.7731 | 649.0025 |
| 9 | 733.0979 | 699.0386 | 686.7834 | 697.1358 |
| 10 | 687.8266 | 714.4132 | 742.4945 | 663.902 |
| 11 | 721.4655 | 895.0441 | 768.608 | 573.1487 |
| 12 | 661.8882 | 688.7924 | 953.2853 | 604.824 |
| 13 | 655.4966 | 957.0129 | 794.933 | 743.9648 |
| 14 | 693.3418 | 724.0904 | 750.6837 | 560.5201 |
| 15 | 705.4609 | 870.7647 | 780.6009 | 838.0944 |
| 16 | 721.7596 | 805.6973 | 640.7506 | 610.3259 |
| 17 | 724.818 | 821.079 | 705.9707 | 826.4395 |
| 18 | 752.718 | 711.6958 | 732.4711 | 730.5785 |
| 19 | 560.2838 | 893.1772 | 792.0628 | 921.6633 |
| 20 | 593.8606 | 583.6317 | 767.9004 | 634.9875 |
| 21 | 708.7815 | 679.9369 |  | 868.1635 |
| 22 | 700.7906 | 679.1547 |  | 775.2338 |
| 23 | 699.6829 | 673.461 |  | 912.0179 |
| 24 | 651.8231 |  |  | 709.2392 |
| 25 | 666.9087 |  |  | 763.1413 |
| 26 | 807.1928 |  |  | 634.4096 |
| 27 | 619.803 |  |  | 781.0376 |
| 28 | 590.0137 |  |  | 722.1548 |
| 29 | 693.0162 |  |  | 879.6251 |
| 30 | 845.6506 |  |  | 619.4911 |
| 31 | 593.2832 |  |  | 830.7531 |
| 32 | 749.1744 |  |  | 764.6139 |
| 33 | 628.3343 |  |  | 747.2454 |
| 34 | 620.6513 |  |  | 889.4652 |
| 35 | 649.5317 |  |  | 902.5979 |
| 36 | 640.1505 |  |  | 988.6124 |
| 37 | 561.1228 |  |  | 847.2671 |
| 38 | 661.4965 |  |  | 831.2605 |
| 39 | 654.7462 |  |  | 664.2366 |
| 40 | 681.5488 |  |  | 893.7769 |
| 41 | 610.8421 |  |  | 845.3857 |
| 42 | 802.9599 |  |  | 813.9316 |
